# Modeling IN out-of-hospital emergency medical services—a scoping review of approaches and applications

**DOI:** 10.3389/fpubh.2026.1825916

**Published:** 2026-06-25

**Authors:** Florian Zahorka, Christoph Strauss, Michael Schmid, Gudrun Wallentin, Philipp Dahlmann

**Affiliations:** 1 Department of Geoinformatics, Paris Lodron University, Salzburg, Austria; 2 Institute of Modeling and Simulation, Eastern Switzerland University of Applied Sciences, Sankt Gallen, Switzerland; 3 Faculty of Applied Healthcare Sciences, Deggendorf Institute of Technology, Deggendorf, Germany; 4 Faculty of Health, Medicine and Life Sciences, Maastricht University, Maastricht, Netherlands

**Keywords:** ambulance location and relocation, ambulance routing problem, emergency medical dispatch, EMS—emergency medical services, healthcare operations management, paramedic management, prehospital/EMS, simulation optimization algorithms

## Abstract

**Introduction:**

Out-of-Hospital Emergency Medical Services (OHEMS) play a critical role in providing timely care for patients experiencing acute medical emergencies. Traditionally focused on rapid response and transport, OHEMS are increasingly evolving toward a more comprehensive role within the healthcare system. This scoping review aims to map existing modeling approaches, identify research streams and to examine whether recent developments in practice can also be found in the modeling literature.

**Methods:**

Following the PRISMA extension for Scoping Reviews, a structured search was conducted in PubMed, Google Scholar, Semantic Scholar, and IEEE. We included English-language case studies and review articles published between 2010 and 2024 that addressed modeling, optimization, forecasting, or decision-support approaches in out-of-hospital emergency medical services. Studies were classified using the Emergency Care Pathway framework, with additional extraction of methodological approach, data source, geographical context, performance indicators and implementation perspective.

**Results:**

We included 150 methodological case studies and 33 review papers. Location and relocation problems were the most frequently addressed topics, and response time served as the primary model performance evaluation indicator. The analyzed literature showed increasing methodological diversification, including simulation-optimization, online and real-time optimization, decision support systems, machine learning-based forecasting, and GIS-supported spatial analysis. In contrast, workload-aware models and equity-oriented approaches have also emerged, although they remain less prominent. A limited but growing number of scholars provide model code to support reproducibility and practical uptake. Yet, cooperation between modelers and practice remains limited. Regarding changes in the provision of OHEMS care, results of this study indicate that these have not yet been reflected by modelers.

**Discussion:**

Evidence by this study indicates that OHEMS modeling has evolved from predominantly static location and coverage models toward more dynamic, data-driven, real time decision support and to some extent implementation-oriented approaches. However, many models still conceptualize OHEMS primarily as a rapid response and transport system. Derived from the review findings, we propose the incorporation of a more dimensional framework into modeling approaches that considers finance and public health structure as well as expected quality. Further, based on the notion of Right Time, Right Care, and Right Place, implementing clinically meaningful time metrics, alternative response options, workload and staff constraints, and care pathways beyond transport to hospital may prove useful.

## 1 Introduction

Emergency medical services (EMS) worldwide have traditionally focused on ensuring a rapid response to victims of sudden cardiac arrests, severe traumatic injuries, and other critical health issues (1). According to the WHO, timely response by an ambulance, appropriate care by involved professionals, and swift transport to a suitable hospital are considered major pillars of out-of-hospital emergency (OHEMS) care today (2).

In recent years, providers have advanced their role within emergency medical services practice. There seems to be an increasing understanding of OHEMS to become an integrated part of the healthcare system, in which emergency management, public health, and safety, and the healthcare system are combined (3). This development was encouraged by the need to deploy scarce resources more effectively and primarily driven by two factors: rising operational costs and a growing call for alternative care paths beyond hospital transportation (4, 5). According to the US Emergency Medical Services Office, EMS clinicians today may be considered a critical component of emergency management and community healthcare practitioners. In this role, they often discover healthcare crises in the first place and need to act appropriately (6). In response to the growing demand for new approaches in OHEMS amongst scholars, Böbel et al. (5) suggested an adapted understanding of the existing WHO EMS framework. While traditionally only considering scene, transport and facility, the authors use the example of Copenhagen EMS to advocate (i) the integration of EMS into primary care and public health, (ii) patient-centered strategies such as a single point of access or alternative response options beyond ambulances, and (iii) the use of technology to enhance care coordination.

Academic modelers in the field of OHEMS have studied various optimization problems for over half a century. As shown by Bélanger et al. (7), Reuter-Oppermann et al. (8), Basar et al. (9), Neira-Rodado et al. (10) and Garcia and Marín (149), a wide range of approaches have been developed over the course of almost half a century. A commonality among such reviews was to categorize most planning and management approaches following a strategic, operational or tactical but mostly time and location-based perspective. Researchers frequently focused on the advancement of methodological aspects. Here, the underlying assumption was, that systems perform effectively when they achieve a single, predetermined performance criteria—most prominently response time. Originally derived from emergency medical research, response time became a core performance indicator globally, significantly influencing modeling approaches (11, 12).

In 2017, Aringhieri et al. (13) published a comprehensive review of state of the art approaches in modeling. The authors suggested a shift in perspective, calling scholars in the field to advance their perception on the management of OHEMS systems. As a synthesis, they introduced the Emergency Care Pathway (ECP) and categorized studies into location, relocation, dispatch, routing, interaction with national health services, evaluation and validation, forecasting, and workforce management (13) aspects. The authors emphasized the interconnectedness of optimization approaches, stating that modelers needed to consider that “decisions taken in one step of the ECP can affect decisions in subsequent steps of the ECP”. Their approach signified a departure from sole methodological perspectives toward a process-oriented aim. Drawing on equity, uncertainty, the first-hour quintet, and risk aversion metrics, they also offered novel performance indicator perspectives. By pointing out these aspects, the authors advocated for a broader, more differentiated approach that requires to integrate a systems perspective.

Another aspect mentioned in the study concerned a growing diverge between OHEMS agencies self-perception and modelers aims (13). One reason was found in the lacking adoption of a holistic outcome-based approach that included the entire patient’s journey rather than, for example, a sole location optimized aspect. This finding reiterated the question whether implementation remains a relevant aspect in OHEMS modeling. A study by Brailsford et al. (14) conducted already in 2009 provided evidence of the low implementation rates of healthcare modeling in general. Their explanation considered the fact, that research grants often value experimental approaches over practical applications and low formal cooperation between academia and practice. Overall, the question on the level of partnership between modelers and practice remains unresolved.

Based on the above-mentioned developments and existing challenges, i) changes in the provision of OHEMS care, ii) introduction of a holistic modeling perspective through the ECP, and iii) a perceived low implementation of academic approaches, the question arises, what the current state of modeling approaches in OHEMS looks like. Departing from more than a decade of collaboration with OHEMS practitioners (15, 16) the following primary research question guided this review:

- What is the current state of modeling approaches in the field OHEMS?

To add additional analytical value to existing reviews [i.e., by (7, 8, 13, 17–20)], we formulated the following literature-guided subsidiary questions:

- What research streams can be identified based upon the Emergency Care Pathway concept and how do recent approaches evolve?
- To what extent do modelers address transferring optimization into OHEMS practice?
- What advancements can be observed regarding evaluation and outcome metrics?
- How is the changing role of OHEMS as an integrated healthcare provider integrated into the modeling practice?

Considering the overall aim for this review, we structured our review upon the work of Aringhieri et al. (13) and their Emergency Care Pathway. Core to their work was the implementation of a more holistic modeling perspective. In this respect, the influence of response time to become the main performance indicator for OHEMS practice and modeling is addressed. In addition, the integration of Brailsford’s (14) practical implementation perspective allowed to investigate how the interdependency between academia and practice has developed. Finally, thorough consideration whether recent changes in the provision of care by OHEMS providers have been reflected by modelers were considered helpful to guide future modeling approaches.

The remainder of this article is structured as follows. Chapter 2 describes the methodological approach undertaken for this scoping review. Chapter 3 depicts current modeling approaches in OHEMS based upon the Emergency Care Pathway. The focus was to present recent or novel approaches. A discussion of results is conducted in Chapter4, including remarks on response time origins and changes in the provision of the OHEMS care model. The supplements of this paper include the data extraction template, search protocols, results tables and links to repositories for model replication.

## 2 Methods

### 2.1 Design

A scoping review as outlined by Peters et al. (21) was undertaken. The PRISMA extension for Scoping Reviews was followed accordingly. This study was pre-registered under^1^. In contrast to a systematic review, the aim was to gain a broad overview, map the existing literature, identify streams of research, and detect possible gaps to highlight potential future directions (21). Thereby, quality and methodology rigor of the studies were not systematically assessed.

### 2.2 Search strategy

As suggested by the JBI committee for scoping reviews, the research framework was based on a population, concept and context (PCC) scheme (22). The population of interest was “Out-of-Hospital Emergency Medical Services”. The concept of interest was defined as “modeling approaches considering the management of OHEMS”. The context was related to “OHEMS planning, modeling, optimization and simulation”. Based on the PCC, the respective search strings were generated. We included published peer reviewed journal articles, as well as conference proceedings published between 2010 and 2024, which in English language only.

For the purpose of this review, a modeling approach was defined as a formal computational, mathematical, simulation-based, statistical, geospatial, or algorithmic representation of an OHEMS planning or operational problem. This included, but was not limited to, mathematical programming, simulation, simulation-optimization, queueing models, Markov decision processes, machine learning, GIS-based spatial analysis, forecasting models, and decision support systems. Studies were excluded when they used the term simulation solely in the sense of medical training or education, when they modeled only in-hospital emergency department processes without an OHEMS interface, or when the modeled system referred to military evacuation, police, fire services, or other emergency services without a specific OHEMS component.

Following the scoping review guidelines, Table 1 itemizes the inclusion and exclusion criteria.

**Table 1:** Inclusion and exclusion criteria.

Inclusion criteria	
Date	Evidence published from January 2010—December 2024
Setting	Simulation/modeling approaches in emergency medical services
Population	Out of hospital emergency medical services
Concept	Modeling approaches considering the management of OHEMS
Context	OHEMS planning, modeling, optimization and simulation
Study type	Case studies or reviews
Language	English
Exclusion criteria	
Study setting	Modeling interventions other than emergency medical services (e.g., Military Medevac, Fire, Police)
	Modeling patient flows in emergency departments

An initial database search using two databases (Pubmed and Semantic Scholar) was conducted to gain the key topics and develop search strings. It became clear that databases other than PubMed handled the search string “ambulance” better than “emergency medical service”, so we used the former. Subsequently, a sensitive search strategy was undertaken to return an extensive body of literature (22). The search included a thesaurus, as well as free text search. Boolean operators were used to combine the search strings. OVID was used to track the various combinations of medical subject headings (MESH) and keyword search in PubMed. The sensitive search was conducted in four databases, namely: PubMed, Google Scholar, Semantic Scholar, IEEE. To complement findings, selective citation searching was implemented subsequently. All search results were recorded in Herzing’s Publish or Perish for Windows (23). An example of the search is provided in Table 2, a detailed search protocol for all databases can be found in Supplementary material S2.

**Table 2:** Excerpt of search strategy.

Database	Sample search term	Sample results
Pubmed	Emergency medical services [MESH] AND system analysis [MESH]	82
	Emergency medical services [MESH] AND operations research [Mesh]	342
Google scholar	“ambulance” + “Agent based modeling” OR “operations research” OR “mathematical programming” OR “linear programming” OR “monte carlo” hospital	262
Semantic scholar	“ambulance” AND “simulation” AND “optimization”	165
IEEE	[“ambulance” AND(“Agent based modeling” OR “operations research” OR “mathematical programming” OR “linear programming” OR “monte carlo” OR “discrete event simulation”)]	56

Medical subject heading (MESH) were used for categorial search. An extensive list of search strings can be found in Supplementary material S2.

### 2.3 Screening process

**Figure 1:**
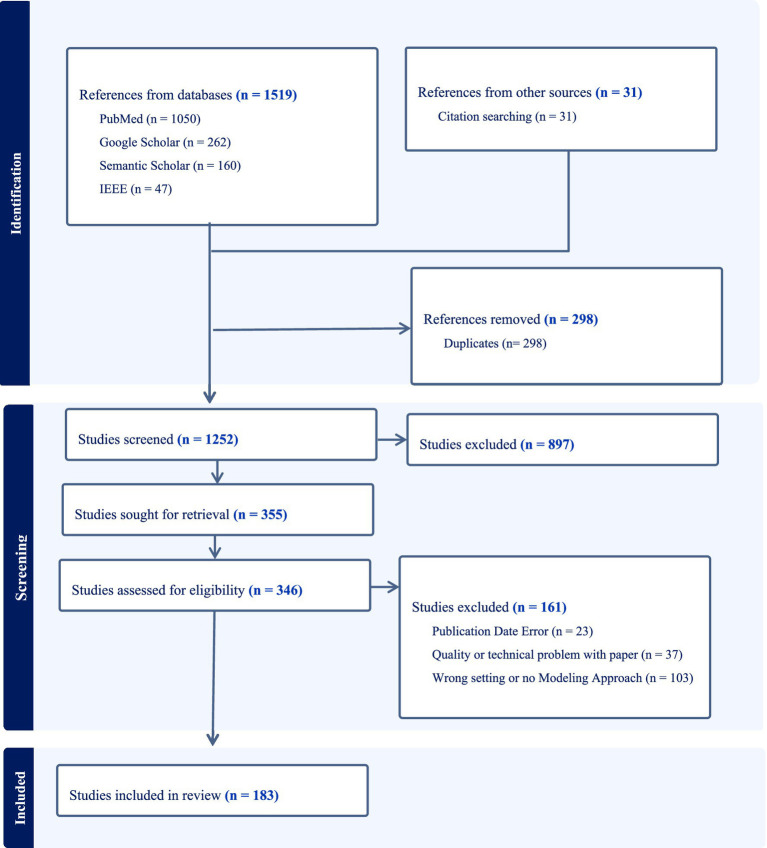
Search results according to prisma-scoping review extension.

The literature search yielded 1,550 studies. After removing 298 duplicates, 1,252 records remained for abstract screening. Each abstract was screened by one reviewer separately. Following this stage, 897 records were unsuitable and got excluded. Out of 355 studies sought for retrieval, 346 studies were successfully retrieved for full text assessment. Next, a criteria based eligibility assessment was undertaken by two reviewers independently. Reasons for exclusion were noted using Covidence where the other researcher checked on the rationale of each excluded study. During this full-text assessment, 103 articles were excluded due to incompatible modeling settings, and 23 because of publication date inconsistencies. We also found another 37 papers had to be excluded for technical or quality-related issues. Within this category “technical or quality related issues” we attributed 26 studies due to initially not recognized invalid article links or access was limited due to subscription issues. Another 11 studies showed problems regarding insufficient language quality, works turned out to be rejected articles, represented a thesis and other grey literature. Consequently, 183 publications were included in the final sample for this scoping review. The search and selection process is illustrated in Figure 1, following the PRISMA Extension for Scoping Reviews (PRISMA-ScR).

### 2.4 Data abstraction

Since our initial search already revealed many relevant studies, we aimed to iteratively create and adapt a comprehensive extraction template (see Supplementary material S1). This template systematically captured context and type, methodological focus, simulation aim, data source and geographic region, data source and implementation focus of the study. Collaborative data abstraction was performed using the Covidence Platform, allowing researchers to assign papers, review their selections and resolve possible conflicts (24). As a contrast to the commonly used operations research framework, which distinguishes between strategic, operational and tactical levels, we based our structure upon the ECP framework developed by Aringhieri (13). The respective classification procedure was mainly derived from the methods section of each study. Our workflow tasked researchers to make the assignment at this stage of the article extraction but allowed to revise the selection at any given time or add multiple selections. Besides this multiple-choice selection, a second single choice selection regarded the primary of the respective approach. This category was used to help determine the main aspect of a study within the ECP. For the subsequent analysis, each study was classified and only counted based on its primary focus. If additional steps of the ECP were addressed, these were recorded in a separate column. As an example, the study by Da Ros (25) was assigned and counted towards the location and relocation category, although dispatching and routing as well as evaluation and validation were also addressed and noted accordingly (see Supplementary material S3). It bears mentioning to the reader, that the main aim of the ECP was not to impose strict categorical boundaries but to acknowledge the interconnectedness of studies despite their topical focus.

### 2.5 Data analysis and synthesis

After completion of data abstraction, the included studies were analyzed using Microsoft Excel PIVOT charts. Descriptive analysis was performed on different characteristics using the categories provided by the template. This step was followed by a spreadsheet based weekly in-depth discussions amongst three researchers. To examine temporal developments in model evaluation, studies published from 2018 onwards were assessed in greater detail with specific regard to the reported performance indicators and key methodological aspects. The year 2018 was selected because it follows the publication of the review by Aringhieri et al. and therefore allowed us to examine whether more recent studies introduced evaluation metrics beyond those emphasized in earlier literature. This analysis was descriptive and was not intended as a formal quality appraisal, but rather as a means of identifying patterns in how recent OHEMS models evaluate system performance differently. In order to establish a concept map of state-of-the-art methods and research streams within the ECP, we developed interpretive analytical groupings to structure the descriptive synthesis of heterogeneous modeling approaches. The respective clustering was initially directed by chronology and frequency of appearance. Afterwards the review team discussed recurring patterns among approaches and marked studies relevant to illustrate a distinction within the streams. The guiding questions were, what approaches appear frequently, what directions can be identified and what exceptional approaches can be identified.

Furthermore, the degree of practical implementation of OHEMS modeling case studies was analyzed, building on the perspective previously introduced by Brailsford (14). Finally, in terms of model replicability we searched in studies for indications of repositories and published models and screened their functionality.

## 3 Results

### 3.1 Characteristics of included studies

**Figure 2:**
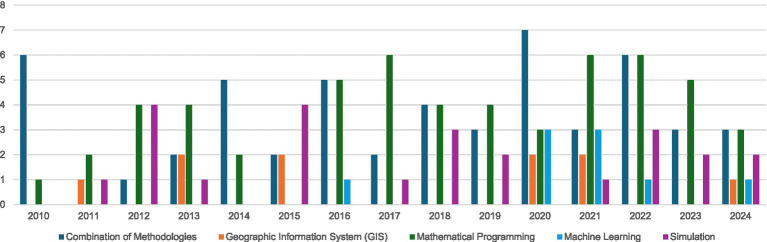
Temporal development of OHEMS modeling methodologies based upon the main approach.

A total of 183 publications from more than 50 different journals, as well as conference proceedings were included in the final analysis. These studies can be further distinguished into 150 (82%) methodologically based case studies and 33 (18%) review articles. In terms of annual publication frequency, a steady increase from 2010 to 2022 (from 8 to 18 annual publications) followed by a decrease until 2024 (13 publications) can be observed (see Figure 2). Case studies mainly addressed European OHEMS—58 (39%), followed by those conducted in Asian systems—36 (24%), North-and South America—35 (23%), South Asia and Oceania—12 (8%), and Africa—6 (4%). Three case studies were conducted using self-generated data without a geographical association. Most methodological case studies implemented a mathematical programming approach (73 publications), followed by a combination of methodologies (64 publications), where simulation-optimization based approaches were predominant. Since 2016, a smaller, but growing number of studies have focused on machine learning (10 publications) as well as Geographic Information Systems (10 publications), see Figure 2.

**Figure 3:**
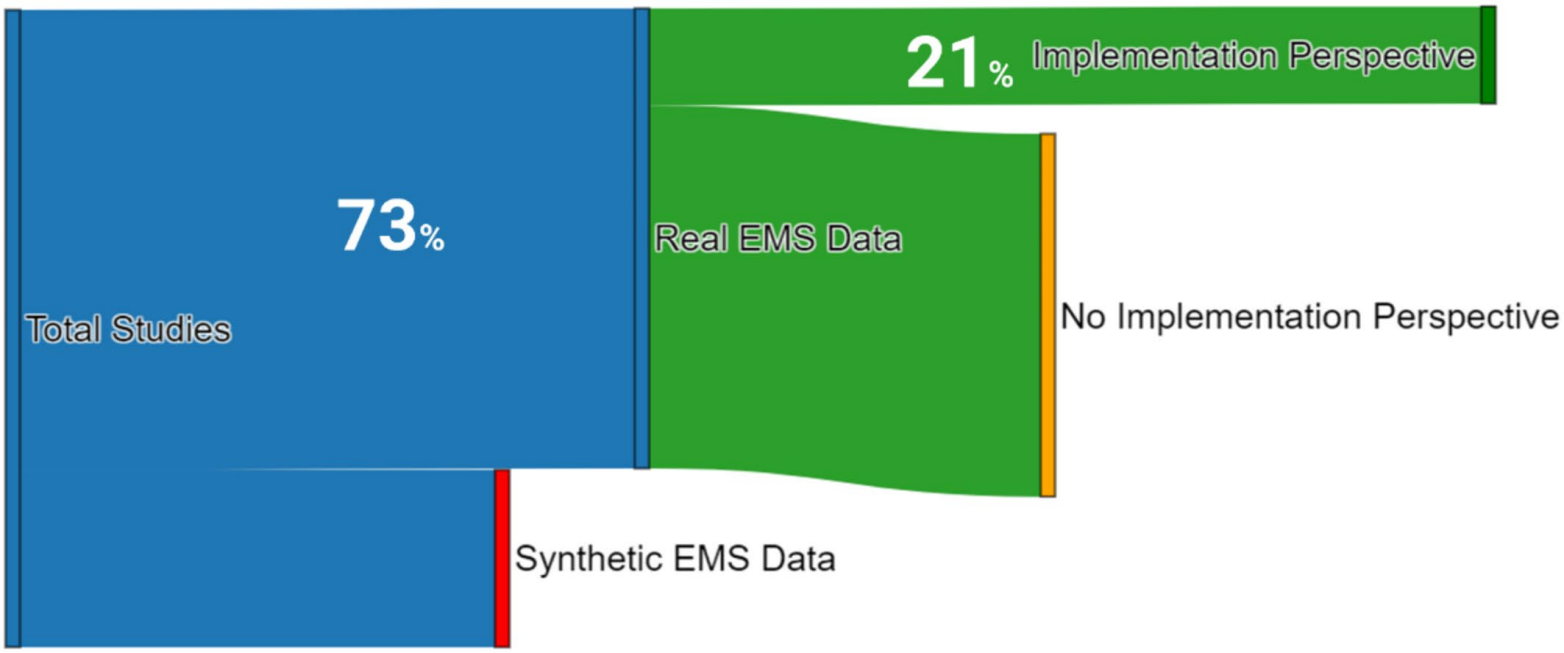
Use of OHEMS data and implementation perspective.

A common approach was to model a specific aspect of the ECP, notably the location of stations and ambulances or the redeployment of vehicles. In such cases, we analyzed the use of actual OHEMS and their primary data source: 109 (73%) studies utilized data from real OHEMS systems, compared to 41 (27%) that used synthetic, self-generated data (see Figure 3). Real OHEMS datasets frequently included station locations and number of ambulances and could also encompass historical incident locations, urgency levels, population distribution and unit staffing. Of the studies using real OHEMS data, 23 (21%) indicated forms of implementation. Where practical implementation was reported, it most commonly consisted of providing modeling results to policymakers and practitioners (26, 27). In a majority of papers, however no explicit implementation or follow-up was reported. A small number of studies described explicit form of ongoing cooperation. For instance, in their multi-year research, Da Ros et al. (25) stated a comprehensive form of exchange with practitioners to develop a decision support system for dispatchers and policymakers. Similar theory-practice exchange was reported in Xu et al. (27) or Dibene (28).

**Figure 4:**
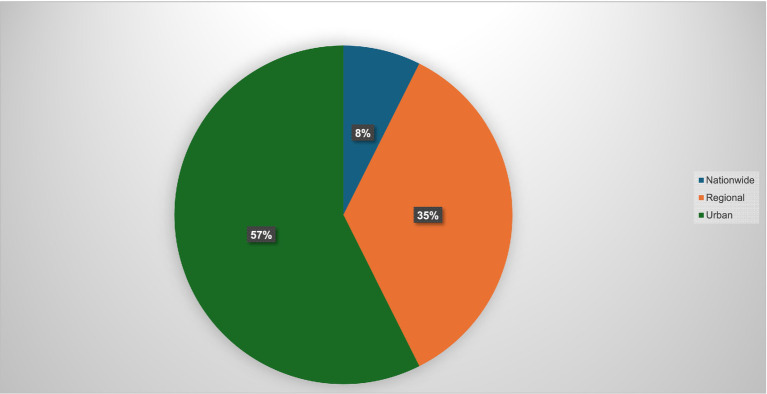
Addressed geographical context of case studies.

Of the publications using real data, 62 (57%) considered an urban context—most were set in cities or metropolitan areas (see Figure 4). This was followed by 38 (35%) studies addressing regional contexts such as statewide or mixed urban and rural. Eight (8%) studies addressed a nationwide context, most of which addressed air ambulance modeling (29–31) Jánošíková (32) provided a nationwide mixed urban–rural ground-based OHEMS optimization model. This appeared to be the only publication creating a comprehensive model on a national scale for the Slovak Republic, spanning across 50,000 km^2^, serving more than 5.4 million citizens.

Regarding model replicability and reproducibility, seven publications provided full or partial access to their code, which amounts to about 5% of all case studies. Supplementary material S4 shows links to repositories and the respective models. Two studies provided an interactive visualization of only their results.

### 3.2 Modeling approaches along the emergency care pathway (ECP)

Findings from this research were structured along the ECP, as introduced by Aringhieri (13). Each step of the ECP is addressed in a separate subsection:

1. Location and relocation
2. Dispatching and routing
3. Forecast
4. Evaluation and validation
5. Workforce
6. Interplay with healthcare system

**Figure 5:**
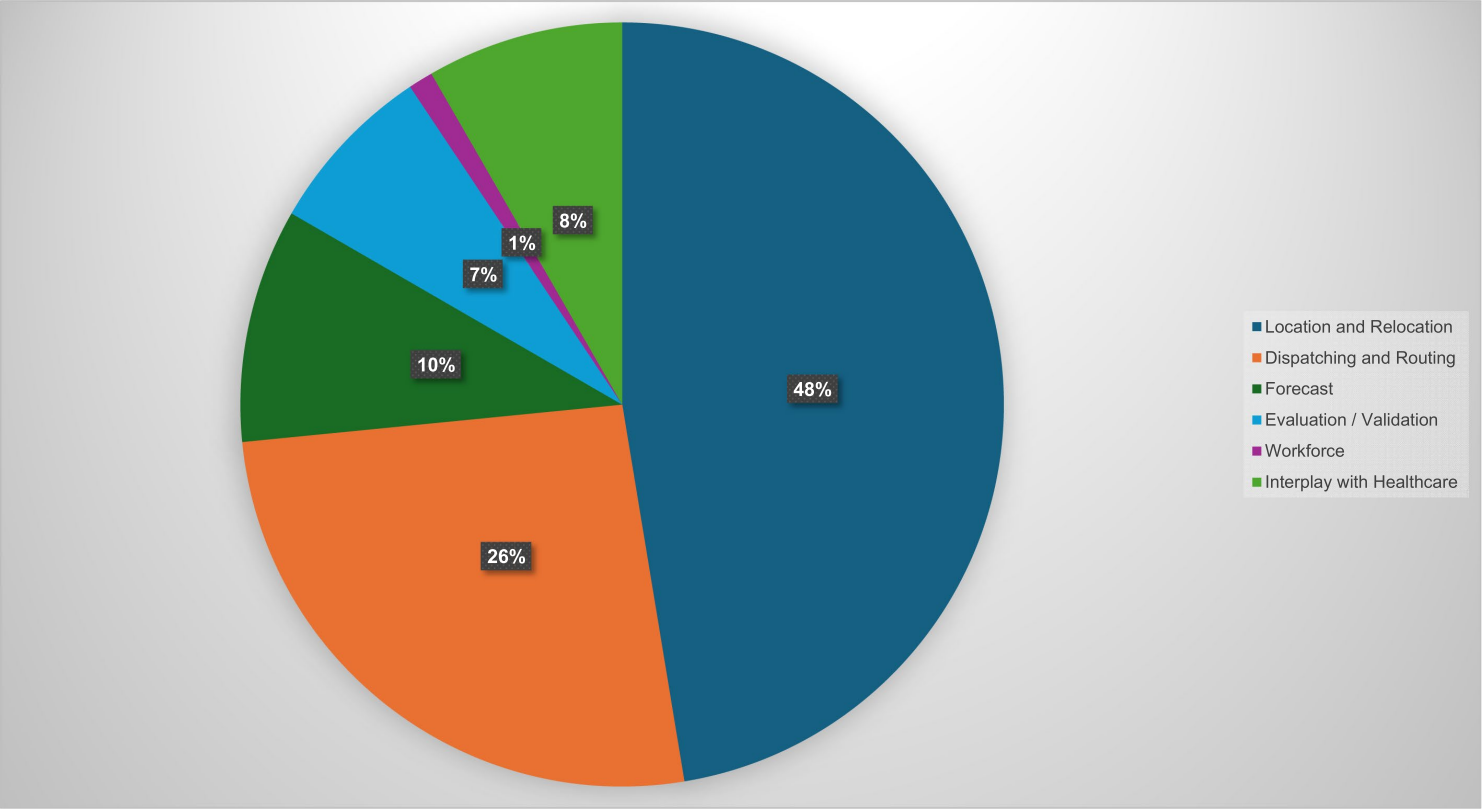
Distribution of case studies along the steps of the emergency care pathway developed by Aringhieri (13).

Figure 5 shows that 91 (48%) of all case studies addressed Location and Relocation problems, followed by 50 (26%) studies addressing dispatching and routing 19 (10%) considering forecast and demand studies, 16 (8%) dealing with interplay with the healthcare system, 14 (7%) addressing evaluation and validation of models, and two papers (1%) considering workforce-associated-models. 43 out of 183 (23%) studies addressed more than one out of the 6 steps above. Thereby, the connection between location and relocation and dispatch and routing was dominant (16).

### 3.3 Location and relocation

Location and relocation problems address the optimal placement of stations and assignment of units. Corresponding models aim to allocate resources efficiently under given constraints and support system adaptation. A consistently high number of review papers and case studies in this area has been observed since 2010. For a concise differentiation of location problems we refer the reader to Basar (9), who created a taxonomy for OHEMS location problems and differentiates problem type, modeling approach, and solution. Authors (9, 33–40) provide an overview of static location set problems to maximize coverage (148), the threshold based maximum coverage location problems, the double standard or backup coverage model to maximize coverage of demand points using two time thresholds, as well as the dynamic maximum expected coverage location problem, which introduced stochastic demand. These models often address a rather strategic planning levels and can provide ex-post suggestions for optimal ambulance stationing and assigning vehicles. Becker et al. (18) provide a recent overview of dynamic location problems, which address the problem of optimal redeployment or relocation of idle ambulances. Their approach can be especially helpful at a tactical/operational level, when dispatchers need suggestions about where to send ambulances after completing a call and whether coverage by an outside ambulance is indicated.

#### 3.3.1 Advancing location relocation models

Based on the interpretive synthesis, three broad research streams were identified in the location and relocation path: studies extending or applying established optimization models, studies combining optimization with simulation, and studies developing decision support systems. First are studies that use and advance an established methodology or pursue application to a previously unused context. In earlier works such as (34–39) researchers focused on solving a deterministic single-domain problem, e.g., locating stations resource allocation. As illustrated by the example of Hammami et al. (40) the integration of stochasticity to better address real world uncertainty gained importance over time. The authors modeled a two-tiered OHEMS system (ALS / BLS ambulances) using a chance constraint programming approach. Recent approaches also show a growing interest in bi-or multi-objective studies, considering, for example cost-effectiveness and increasing survival rates (38, 41–46). As seen in Zhang et al. (47) in 2024, the authors developed a comprehensive Multiperiod Capacitated Facility Location Problem with Maximum Travel Time and Backup Service (EMSLSP). In total, they incorporate six different objectives ranging from travel time, service capacity, dynamic demand for backup coverage, and the number of ambulances assigned to a station. Due to increasing complexity or the extent of the study area computation becomes time consuming. Particularly relevant to relocation issues, this computational challenge is addressed by implementing metaheuristics such as particle swarm, genetic algorithms, tabu search were implemented to find near-optimal solutions within an acceptable time frame (48–50).

#### 3.3.2 Combination of optimization-simulation

The second recent research stream considers the use of combined methodologies. Here, optimization-simulation approaches are used, where the simulation aspect aims at mostly at validating optimization results. Frichi et al. (51) see the advantage of simulation in its level of detail, the possibility to deal with several sources of uncertainty, and accurate prediction of performance indicators. According to their classification, simulation approaches can be categorized into i) Discrete event simulation, ii) Continuous Simulation, iii) Hybrid Simulation, iv) Monte Carlo Simulation, and v) Agent based Simulation. Discrete Event Simulation is the dominant simulation method used in OHEMS modeling, as seen for example in (15, 26, 32, 51–56). Integrating these different approaches into a generic framework, Berchi et al. (57) were the first to suggest a five-step methodology:

1. Analyze data involved in the process to compute the needed input data
2. Solve a static and deterministic optimization model to provide a solution
3. Evaluate the obtained solution through a simulation model
4. Propose the results to EMS planners to gather feedback and repeat steps 1–4
5. Integrate the solutions to improve and guarantee continuation of service

Methodological advancements have already modified the framework. For instance, Lee et al. (58) advanced step 2 by including stochasticity in the optimization model to gain the optimal location of air ambulances and trauma centers. Zang.et al. (47) implemented a set of spatio-temporal demands already in the deterministic model to improve validity *a priori*. Authors (32, 47, 50, 53–56, 59–62) provided recent examples with a variety of enhancements but basically follow steps 1–3. Besides using simulation for subsequent validation purposes, authors like Schjølberg (50) created an iterative solution where the simulated output (response time) served as a fitness function for a subsequent optimizer, which in turn provided input for further adapted simulation runs with new locations. Ong et al. (63) developed *OpenEMS,* a versatile open-source framework for simulation-optimization. They published their model, which can be used for optimal stationing of ambulances. Strauss (64) also developed an open-source model for dynamic ambulance redeployment simulation tool named *AmbuSim*. Their model used publicly available data from San Francisco EMS allowing the implementation and evaluation of different redeployment strategies such as a dynamic MEXCLP, an expected response time model, or demand forecast using reinforcement learning.

#### 3.3.3 Online real time decision support systems

A third, recently re-discovered research stream concerns the development of Decision Support Systems (DSS) for OHEMS management. Models aim to analyze the system status to suggest solutions for decision makers or dispatchers on a strategic, operational, or tactical level. Aringhieri repeatedly (56, 65) argued that online optimization seemed suitable for OHEMS planning, especially in fields of relocation, dispatching and routing because they operate within dynamically changing environments characterized by intrinsic uncertainty. Rather than following predefined stochastic based modeling suggestions, commonly referred as offline optimization, the author concluded that timely (or even real time), computationally feasible decisions could be beneficial, even if they are not globally optimal. In this context, “online” refers to optimization procedures that update recommendations based on the current system state, for example when a new incident occurs, a vehicle becomes available, or demand conditions change. Recently, computational as well as methodological advancements have led to the further pursuit of this stream, especially by the integration of a real-time optimization component (25, 49, 56, 66). As illustrated by Bhatia et al. (66) such systems take into account the site of occurrence and seriousness of incident, current and expected traffic situation, presence of ambulances and their actual location, expected future emergencies from a prediction component and hospital capacities and specialties. The authors implemented an Negamax algorithm, that allowed multi-factorial decision making in real time considering all these specifics.

In 2024, Da Ros et al. (25) published results from an ongoing study on a decision support system for an entire region in Northern Italy. Their DSS contained a dashboard, a message-passing component for linking user actions, and a distributed task queue, which runs the simulation and optimization for the decision maker. Since the discrete event simulator functions as a digital twin, it allows dispatchers to analyze the current situation, provide and model suggestions for interventions, and to evaluate dispatch decisions. A Pareto front visualizes the occurring trade-offs between conflicting interests such as fair coverage, population coverage, surface coverage or second ambulance distance. In this regard a Late Acceptance Hill Climbing multi-objective local search algorithm was implemented, creating a candidate solution, comparing it to the reference and to the next in history to decide whether the solution is disregarded or not. Dispatchers are then provided with suggested solutions and the possibility to simulate the effects.

Andersson developed a Maximum Expected Performance Location Problem with Heterogeneous Regions Model as a decision support tool for strategic ambulance planning. Their model aimed to evaluate the consequences of system adaptations such as hospitals closures, adaptions in fleet management, or time variation of operating hours to help inform what consequences may arise from these changes (67).

The common goal of DSS may be split into matters of what vehicle to send to occurring incidents (=ambulance dispatching), where the units should go to (=facility selection) and what they must do next, after a mission is completed or when they become idle (=Ambulance Redeployment/Relocation) (68). As such DSS may be particularly relevant in high-load or congested systems, where dispatch, relocation, and routing decisions are highly interdependent. Yet, the implementation of multi-unit systems, different contradicting objectives and the continuous stream increases complexity significantly compared to deterministic models and raise an important question, what purpose the DSS pursues.

#### 3.3.4 Geographic information systems

The integration of geographic information system (GIS) resources in locations models has become an important part in modeling, due to its inherent spatio-temporal perspective. Besides the main ability to visualize for example coverage areas (50, 69–71), GIS allows for systematic collection, editing, and analysis of data. Common OHEMS resources are incident locations (70, 72–74), ambulance or facility locations (63, 72) or real time GPS based tracking of lights-and-sirens travel (75). Besides that, GIS can also be used to compare socio-demographics in demand areas, identify care gaps and visualize socio-economic inequalities as demonstrated by Guo (76). Another perspective on GIS is the development of routing algorithms, that enable a more realistic perspective on coverage (15, 63), especially when considering street networks, traffic data and weather variations (77). Preprocessing steps such as geocoding, map matching, outlier removal, demand aggregation, and the construction of time-dependent travel matrices therefore become important parts of model development. In relation to combining GIS and DSS, one of the possibilities is to use exact real-time incident locations, real time travel speeds, traffic, weather and smart city data, whereas static or dynamic location models often grid based demand aggregation to reduce computation time.

### 3.4 Dispatch and routing

Dispatching and routing address the issue of assigning the appropriate vehicle type and providing the optimal path to an incident. There is an inherent linkage between location and relocation decisions on the one hand and dispatch and routing on the other. Nevertheless, one characteristic of dispatch and routing problems is that operational decisions are required immediately, depend on the current system state, and include high levels of uncertainty (13). Therefore, the implementation of real time online (dispatch) decision support systems may provide valuable support for the dispatcher to determine what unit they should assign to a call. An overall goal is to provide a stable and reliable system and keep a high level of availability for further or rare exceptional events.

#### 3.4.1 Dispatch strategies

Dispatch strategies may be categorized into straightforward and more advanced ones as shown in Table 3. For example, the application of a basic closest-idle strategy can be considered an intuitive and widespread approach for both OHEMS providers and researchers (13). Other studies suggest alternative methods such as zone dispatching (16), priority dispatch (32, 78), “human-expert-based” dispatch (79) or “algorithm-aided” dispatch (31, 57, 64, 77–81). It is important to note that “algorithm-aided dispatching” may be interpreted differently between OHEMS professionals and researchers, since the use of predefined, standardized protocols for emergency calls and dispatching can already be considered a form of algorithm-aided dispatch.

**Table 3:** Dispatch strategies in OHEMS.

Strategy	Description	Example
Closest idle	Send the closest available idle unit	Currently used in most EMS systems (13)
First in first out	Send units according to idle time	
Zone dispatching	Send units according to an area assignment plan	(16)
Priority dispatching	Send units according to the call urgency and select the appropriate vehicle type. Incorporates closest-idle but next-best (=suitable for call urgency)	(32, 68, 78)
Expert-based dispatch	Let system agent (eg. dispatcher) decide a suitable strategy	(79)
Algorithm aided dispatch	Integration of queuing algorithms Markov decision process, machine learning to aid dispatch process, incorporates aspects such as risk-assessment, survival or user abandonment probability or crew-workload	(25, 49, 56, 66, 80, 81, 93, 114)

Another aspect in regards to dispatch strategies considers the creation of digital twins as shown in Wolf et al. (82). A smart city online optimization approach may provide immediate support for the dispatch decision, integrating traffic or weather data. Bhatia et al. (66) incorporated what they called the SMART ambulance system that consisted of data collection and processing, analysis and prediction, resource management and optimization, communication, user interface, and a storage component. Their aim was to optimize dispatching strategies using a game-theory approach with real-time data. Lam et al. (75) developed an open-source tracking and optimization tool. They integrated a customizable dispatch simulator based on a user-defined dispatch policy. The policies addressed ambulance selection by travel time, coverage, or a weighted combination, target hospital selection, and base selection after completion. Their dedicated low-cost solution was developed and implemented in cooperation with an OHEMS provider in Mexico. Roa et al. (49) introduced a preparedness index for online real-time dispatching, looking at priority, system performance, and capabilities of units. They considered preparedness as a valuable index for system performance. Elfahim et al. (81) implemented a Markov decision process using machine learning algorithms to improve dispatching strategies and minimize average response time.

#### 3.4.2 Vehicle routing

Routing is of high importance for dispatch decisions because they involve two main aspects: identifying the optimal route for a unit and deciding which unit to dispatch eventually. The use of lights and sirens runs, changing traffic conditions, and environmental factors such as weather are typically not considered in standard routing algorithms. Torres et al. (83) used different Machine Learning approaches (e.g., random forest) for the realistic estimation of travel times. They trained their model on the different decrements and increments between Google Maps and OSRM travel times and historic GPS-based travel times. Schneider (84) used a simulation tool to estimate the impact of lights and sirens on travel time, using a linear regression model based on historic travel times to provide realistic isochrones/catchment areas. Hojgaard et al. (85) suggested the prediction of travel times based on GPS data and provide an equation for urban and rural OHEMS travel time prediction. Luan (77) introduced a mixed-integer linear programming with semi-soft time windows (MIPSSTW) for improving vehicle routing in highway incidents tackling the problem of complex time varying traffic situations.

Combining routing and dispatching, Aringhieri et al. (56) developed an integrated real-time dispatching, routing, and redeployment policy (DRRP) approach. This simulator allowed real-time management of ambulances, containing a set of online algorithms that implemented strategies such as closest idle, considering that there are enough close bases, setting a cutoff priority queue that stops serving low urgency issues and finally allowing assignment of ambulances even during the redeployment phase. They also included hospital selection policies and different ambulance redeployment strategies. Shin (86) addressed dispatch problems during a mass casualty incident. They developed a finite-horizon Markov decision process addressing what patients are transported to which hospitals and how additional patients.

### 3.5 Interplay with healthcare system

In many cases OHEMS serve as an entry point into the healthcare system. Investigating the interaction between involved actors seems beneficial for a comprehensive understanding of integrated care systems, incorporating prevention and patient-centered care. According to Aringhieri et al. (13) modeling approaches can be categorized into three main areas: those focused on emergency departments, those addressing overcrowding, and those pertaining to ambulance diversion. Each of the three aspects deals with the impact on the availability of OHEMS units.

#### 3.5.1 Addressing system load issues

Pforringer et al. (87) created an agent-based simulation using the web-based information system called IVENA of the city of Munich. They tested a threshold-based emergency department closure policy (simple $\text{Crowding Index}=\frac{\text{current load}}{\text{unit capacity}}$ ) against two time based closure policies. Their aim was to optimize closure policies of either 6–12-24 h based on whether full or 80% capacity was reached. The authors highlight negative consequences of diverting ambulances due to overcrowding: especially in the case of acute myocardial infarction, stroke, or sepsis, ambulance diversion was associated with increased mortality.

Kovalchuk (88) combined a game-theoretic modeling with a discrete-event simulation approach. The goal was to demonstrate the effects of confronting/contradicting stakeholder positions between ambulances, hospitals, drug stores, and health insurance companies. Their early-stage case study considered dispatching and queuing, as well as rejection and rescheduling in the case of acute coronary syndrome patients. The relevance of policy optimization caused by varying demand and overcrowding of hospitals was demonstrated.

#### 3.5.2 Modeling novel OHEMS intervention strategies

A novel approach in this field was undertaken by Maas et al. (89) regarding the treatment of acute ischemic strokes in rural areas. Currently, OHEMS often follow a “drip-and-ship” approach, where patients are transferred to a primary stroke unit and subsequently to a comprehensive stroke center. This procedure often results in a substantial delay in treatment. In their study, a Monte Carlo simulation baseline model of “drip-and-ship” was tested against a “drive-the-doctor strategy.” The new strategy allowed mobile neuro-physicians to travel to a primary stroke unit for intervention, bringing the expert to the patient, resulting in faster treatments. Another example was provided by Phan et al. (90). The authors developed a linear equation-based optimization model for a mobile stroke intervention unit in the city of Melbourne. Equipped with a mobile CT scan the unit was capable of diagnostics and on-scene delivery of medication. Their goal was to find optimal operating parameters such as spatial operating boundaries. Results were presented using an interactive visualization (see Supplementary material S4).

Pilberry (26) created a patient simulation tool for triaging patients between the Great Britain National Health Service emergency medical services dedicated for life-threatening emergencies (dial number 999) the primary care service system dedicated to non-emergencies (dial number 111). In cooperation with the ambulance service provider, they investigated different strategies to determine avoidable emergency department attendances. Their model was published as part of a strategic partnership – the Health Service Modeling Associates Programme.

### 3.6 Forecast

OHEMS providers gather a wide range of spatio-temporal data on call, travel, destination, and treatment. Analyzing these historical data provides insights into demand patterns and may also trigger changes in care provision. Based on a historical understanding, the aim is to predict timely emergency demand and allocate resources accordingly. Aringhieri (13) laid out three distinguishable aspects in OHEMS forecast research: emergency demand, workload, and travel time.

#### 3.6.1 Demand forecast

In a recent study by Manaa et al. (91) the authors implemented different machine learning techniques (CatBoost, Random Forest, Artificial Neural Network, XGBoost) to anticipate future demands and suggest deployment strategies. The authors’ approach was to use spatio-temporal ambulance coverage prediction as a proxy value for demand forecast. Granberg et al. (92) developed a simulation-based prediction of the near-future state of ambulance vehicles. It provided dispatchers with insights into possible future states (30–60 min) and a predicted zonal response time. A novelty was that the engine was able to run multiple replications to capture and visualize various samples of the future state.

In contrast to narrow temporal forecasts, a number of authors developed spatio-temporal prediction models using larger time scales (90, 93–96). State-of-the-art approaches include ARIMA, harmonic regression, trigonometric seasonality, Box-Cox transformation, ARMA errors, Trend with Seasonal components, moving average, support vector regression, and multi-layer perceptron. Rezaei et al. (94) created a multivariate forecasting model that projects 8-h time windows for ambulance station catchment areas with a forecast horizon of 2 weeks. They implemented their model using data from three Canadian cities. In conclusion, the authors consider a low number of incidents and a shorter forecast period (e.g., finer than 4 h forecast resolution) as major challenges in forecasting.

#### 3.6.2 Workload forecast

An example of a workload related forecast was published by Xu (27), who developed a simulation based forecast model for hospital bed availability. Their model predicts the conditions in various emergency departments to understand why some patients get rejected over others. They attempted to integrate specifics from all emergency rooms and allow capacity analysis and demand forecast over the next 20–40 and 60 min.

#### 3.6.3 Travel time prediction

In terms of travel time prediction, Kamal et al. (97) propose a novel algorithm called NextSTMove, capable of predicting future locations of emergency vehicles based on previous locations. Despite the already existing possibility of real-time GPS tracking, this approach may provide useful insights in terms of trajectory analysis and help predict the next position of a vehicle. Overall, a key challenge in forecasting remains finding the suitable resolution for spatio-temporal predictions.

### 3.7 Evaluation and validation

To evaluate and measure the effects of a model, performance indicators are a necessary instrument. These help to answer the fundamental question of whether an improvement can be observed when parameters are changed. In this respect, the definition of relevant outcome factors remains a key aspect. Guided by the common understanding of OHEMS as “fast help,” it appears intuitive that time-based metrics play a decisive role. According to Zaffar (98) maximum coverage, minimum average response time, and maximum survivability are well known metrics. Coverage can be considered a proxy of response time, because it is typically interpreted as the number of incidents or population / areas covered within a defined time limit, for example 15 min, set at a certain threshold, for example 90% of all cases—leading to the widespread “90/15” response time threshold rule (15). A systematic review conducted by Cabral (99) showed that response time measures are frequently used around the world. Target values range from 5 (Taoyuan, Taiwan), up to 30 min (Athens, Greece). Regulations within one country can vary, as seen within Germany: 95% of incidents within 15 min in the state of Baden-Württemberg, 95% within 12 min in the state of Sachsen, a gradual response time from 12 min for densely populated areas to 15 min for sparsely populated areas in the state of Thüringen (100). Besides the intuitive understanding of response time as time elapsed from dispatch to arrival of the first vehicle on scene, there have been many adaptations on “where” and “when” to define the spatial–temporal boundaries of this measurement (12, 15).

### 3.8 Origins and relevance of response time metrics

Within the broader set of time-and location-based performance metrics used in OHEMS research, it is notable that a large proportion of studies ultimately focus on response time thresholds as the primary indicator of system performance. Given the wide range of possible system objectives, including (fair) coverage, workload balancing, patient outcomes, resource utilization and cost optimization found in this review, it raises the question how response time remained its impact as the main performance indicator in both research and operational practice.

To help understand the role of response time, it may be useful to briefly examine the historical development of this standard and its institutionalization within emergency medical service systems. As stated by Al-Shaqsi (12), in 1979 MD Mickey Eisenberg (11) published an article named “Cardiac Resuscitation in the Community”. To the best of our knowledge the author was the first to show a positive correlation between short time and better survival in case of cardiopulmonary resuscitation. Despite his warning, that response time alone does not provide sufficient evidence for successful outcomes, because it “says nothing about when CPR is initiated”, the response time metric became widespread. Subsequent out-of-hospital cardiac arrest, studies confirmed the argument that short response time may save more lives (101–105). The European Resuscitation Council acknowledges the necessity of fast response in situations of cardiac arrest. Nevertheless, the detailed ERC guidelines indicate no recommendation for a single time-based response criterion in OHEMS, but emphasized the so called chain of survival (106). Al-Shaqsi (12) noted, that the widespread adoption of response time as a key performance indicator can largely be attributed to its simplicity and its ease of communication to stakeholders and the public. Ingolfsson (107) suggested that planners must at least consider the following points, when reporting performance measures about their system:

- Should medical or response time statistics be reported?
- What type of response time metrics are reported (average, median, percentiles)?
- Are there different response time standards and reports for call priorities and between rural and urban areas?

#### 3.8.1 The prevalence of novel performance measures

To investigate whether novel response time metrics have been introduced since Aringhieri (13) publication, a selective in-depth analysis of papers from 2018 onwards was conducted. Table 4 provides a summary of different outcome parameters. Of 83 publications, 70 applied time-based evaluation metrics (see Supplementary material S3). Researchers used several response-time variants, for example: mean or median, average, maximum, and multiple-tiered/backup times. Çapar (108) contested response time arguing to put more emphasis on measuring the arrival of appropriate unit on scene. The author suggested a weighted, total response time instead of a “first response time”. Yet, response time still is the most used performance indicator in OHEMS research.

**Table 4:** KPI/outcome parameters of models including references.

Time based	Equity/fairness	Patient/outcome oriented	Efficiency
Response time (15)	Gini-Coefficient (25)	Patient satisfaction (112)	Data Envelopment Analysis (121)
Coverage (98)	Rawlsian criterion (110)	Survival (109)	Ambulance diversion (117)
Survival (109)	Social welfare function (31)	User-abandonment /Willingness to wait (114)	Hospital closure time periods / bed availability (87)
	Bernoulli-Nash (31)	Prioritization of severe incidents (86)	Staff workload (112)
	Iso-elastic social welfare function (31)		System preparedness (49)
	Distance minimization/Spatial equilibrium (111)		

Four studies implemented a survival probability, which can be considered a proxy of medical outcome (49, 54, 79, 109). The underlying assumption is that increasing waiting times for an ambulance are associated with a decreasing survival probability, making survival rate a continuous function of response time. While earlier studies addressed survival exclusively in the context of disaster, two recent studies present non-disaster contexts as well (54, 109). Overall, the relation between survival and response time has only been thoroughly investigated in cardiac arrest cases (13).

Despite the absence of a common framework of multidimensional performance indicators and of equity measures, both horizontally—meaning all demand nodes should be treated equal—and vertically, meaning services should be distributed based on community needs, an emerging number of studies have incorporated alternative measures (25, 29, 31, 110–112). The study by Da Ros et al. (25) implemented a comprehensive decision support system using a multi-objective optimization component. In their understanding fairness minimizes disparities between different regions and integrates the municipal Gini Index into coverage optimization. McHenry based their optimization approach on the inverse care law, arguing that the provision of healthcare equity needs to recognize social determinants as key driver of health requirements and locate services accordingly (113). Grot et al. (110) introduced the Rawlsian criterion, an equity measure which maximizes the coverage of the least-covered demand area. Jagtenberg et al. (31) used a utilitarian social welfare function (SWF) and compared outcomes with other functions to achieve fairness in air ambulance coverage. Their approach originates from welfare economics and combines individual preferences to make a joint decision. Location models mostly optimize towards efficiency. Therefore, a utilitarian social welfare function implicitly favors high demand, typically presented in densely populated areas, and resulting in an imbalanced provision of care. In their model, they compared the SWF with the Bernoulli-Nash, which puts more weight on individuals with low utility values (“no one left behind”) as well as an iso-elastic SWF which creates a continuum between utilitarian and Bernoulli Nash and is more flexible.

Marla et al. (114) added a new evaluation perspective from OHEMS in emerging countries. They addressed the issue of user abandonment when waiting times for ambulances exceed an individual’s willingness to wait. Their simulation-based optimization approach was designed to modify dispatch policies as well as ambulance reallocation to increase the number of calls successfully served.

Ambulance diversion rate, e.g., due to overcrowding, is another frequently used indicator to evaluate OHEMS systems performance (27, 115–120). This phenomenon can often be observed in busy urban healthcare systems or when the restructuring of hospital capacity occurs. Begicheva et al. (112) combined a system dynamics stocks-and-flow model and an agent-based model to evaluate the current OHEMS system of a large metropolitan area. Their model considered time, workload, and patient-based satisfaction metrics.

Another approach measuring system efficiency was implemented by Modh Hassan et al. (121). The authors used a Data Envelopment Approach, which considered efficiency as the maximum feasible output (= distance driven by ambulances) from given input (= operating costs of the ambulance station). Their aim was to identify unproductive ambulance stations and suggest a different allocation of financial resources.

#### 3.8.2 Validation of OHEMS models

A key aspect when developing new approaches is to determine the accuracy of the solutions obtained by the model. Results from this review show, that simulation-optimization approaches have gained importance in location modeling (25, 47, 50, 59). Simulation models may be used for the purpose of validating the optimization model or to integrate stochasticity from a variety of perspectives (incidents characteristics, locations, travel time). Using simulation, modelers aim to reveal optimization issues and to propose alternative scenarios. Because of its flexibility and the possibility to collect system states, discrete event simulation seem to be widely used (15).

Validation is of importance not only for modelers themselves, but also when results trigger practical implementation. Strauss et al. (16) presented insights from a decade of modeling using their discrete event simulation in Switzerland. They extended the methodology-based perspective by integrating close cooperation with domain experts. The authors highlighted that simulation and optimization approaches should go hand in hand with system analysis. Methodological advances should lead to improved planning processes across multiple political levels, increase collaboration between diverse actors, understand feedback, and mitigate unintended consequences. In this respect Berchi et al. (57) was already mentioned, who introduced a the five step methodology for ambulance planning. The authors specifically mentioned external validation in step 4, “to propose the results to EMS planners to gather feedback and repeat steps 1-4”, and iterative, practice-based validation in step 5 “to integrate the solutions to improve and guarantee continuation of service”.

Another aspect of the validation process considers reproducibility and replicability, which will not only help other modelers to understand the approach but also allows to build upon and advance an existing framework. Overall, open model approaches, for example the computational modeling in the social and ecological sciences library, support the development of reusable general-purpose models as suggested by Aringhieri (13). Our review showed that seven case studies partially or fully published their code or visualization (see Supplementary material S4).

### 3.9 Workforce management

Workforce and workload management consider the use of (human) resources and their respective skills in terms of scheduling and rostering. Aringhieri (13) introduced another dimension of workforce management, namely fair composition of healthcare teams to guarantee quality of health services. This approach marks a shift in perspectives from a facility-based to a patient-centered to ensure efficiency and fairness of the delivered services.

#### 3.9.1 Balancing optimization and workload

Overall, reasonable workload and improved responsiveness remain to be somewhat conflicting goals in optimization. For example, all too frequent redeployment decisions might exhaust ambulance crews leaving less time for self-care and further duties (18). To address a fair workload distribution, Catumba-Ruiz et al. (122) implemented a criterion based on the mean number of incidents covered by each ambulance. Zaffar (98) considered equitable work distribution via the standard deviation of an individual ambulance busyness compared to the average business probability. Another approach is to introduce workload restrictions for ambulances, maximizing coverage but also minimizing the number of relocation trips as suggested by Roa et al. (49). In this regard the introduction of threshold-based policies, that limit redeployment missions may help to improve balancing (68). In contrast, Luo (123) limited the maximum number of citizens served by an ambulance in their model using an upper bound. Multiple other studies addressed the issue of balancing workload from a resource perspective. For instance, they differentiate between staff capabilities and vehicle capacity of Basic Life Support (BLS) (124), Advanced Life Support (ALS) (109), or physician-staffed units (69). In such cases the aim was the adequate assignment of units to an incident.

#### 3.9.2 Addressing system load measures

Addressing system load seems to be an important issue when considering workforce management. Andersson (67) presented a case study regarding the effects of removing non-urgent transports from ambulances. Already in 2013 Ingolfsson (107) discussed effects of congestions and questioned whether OHEMS services times depend more on location of ambulances and call addresses or on system load. The integration of citizen rapid responders as well as drones have gained growing attention (68). To optimize staffing in a dispatch and call center Sariyer et al. (125) implemented a news vendor approach. Their goal was to minimize the total costs of expected understaffing and overstaffing to determine an optimal schedule. All these aspects implicitly assume, that high load exhausts crews and should be mitigated.

In contrast, Aringhieri et al. (13) argued in favor of fair composition of healthcare teams that guarantees quality of health services. They considered that routines were essential to provide quality care. In a subsequent work the author introduced a fair distribution policy of emergency department workload by allowing ambulances to reroute non-urgent patients (65).

Considering the goal of OHEMS management to effectively use limited resources, the integration of the workforce perceptive has become part in several of the included studies. In many cases, balancing workload is considered an optimization restriction rather than the primary objective. Incorporating workload seems methodologically beneficial, not only because of the contradiction with response time criteria. Such models provide the possibility to increase equity, practical feasibility and ensure operational acceptance of the model.

## 4 Discussion

Although a formal assessment of study quality was beyond the scope of this scoping review, creating assumptions and simplifications represents a central element of modeling and therefore warrant consideration, as they may substantially affect the validity and interpretation of reported results. For example, by using Euclidean distances, inadequate travel time estimations are returned in the model (126). Also, the clustering of the spatial resolution to centroids or the aggregation of incidents into time periods in favor of computation time may also be misleading. Another typical simplification assumes that all ambulances start from their station (107), which does not hold in urban contexts where follow-up dispatches occur frequently, leaving the team no time to return to its base. The implementation of GPS based real time online decision support systems seems promising to overcome these methodological drawbacks. Optimization models addressing disaster response may be too oversimplifying in case they only optimize for fast transport times (127).

### 4.1 Recent perspectives in OHEMS Modeling

**Figure 6:**
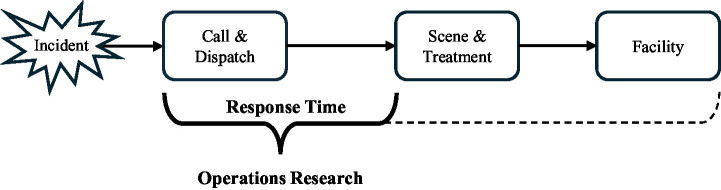
Simplified OHEMS process from incident to facility.

Nearly half of all studies included in this review followed a location and relocation approach. We tried to provide a rationale by showing the medically driven origins that influence current response time policies. Figure 6 depicts the common perception of OHEMS and currently researched areas for modelers. Some scholars implemented advanced solution heuristics due to computational necessity (48–50). Simulation approaches were mainly used for validation purposes, whereas the integration of simulation-optimization has largely found use. Furthermore, due to increasing computational resources, decision support systems have regained considerable attention. The creation of digital twins, online optimization using GIS may advance as a research stream, particularly when real-time or near-real-time operational data are available (49).

In contrast to the above named scholarly advancements, a study conducted by Brailsford et al. in 2009 (14) criticized the lack of implementation perspectives in modeling healthcare. The authors showed that most approaches stayed within academia and did not have any practical implications. Results from our study seem to emphasize a lacking implementation perspective among modeling approaches (Figure 3). Almost 80% did not indicate whether they pursued an implementation perspective, only a few scholars reported on active collaborations with practice. Reasons may be found in the limited access to (open) health-data as well as time-consuming approval processes. An explanation by Brailsford et al. (14) may lie in funding strategies, such as straightforward simulation studies are less favored for research grants than experimental approaches (14). Nevertheless, after a decade of modeling experience in OHEMS, Strauss (16) concluded that systemic coordination and collaboration prove to be more beneficial than sole increase or shift of resources. Established forms of cooperation between academia and practice can be found in several recent publications (16, 25, 26, 63, 75, 95). In the United Kingdom, collaborations between research groups and the National Health Services can be found more frequently. For example, the Health Service Modeling Associates Program represents a training program, specifically designed for health care professionals to acquire operations research techniques (128). Their approach aligns with the WHO Framework for Operations and Implementation Research in Health and Disease Control Programs which asks modelers to conduct a planning, implementation, and a follow-through phase (129).

### 4.2 Relevance of response time

Among the studies analyzed in this research, we found that response time was mainly used to evaluate the implications of the suggested modeling approach. Our analysis showed a considerable variation in response time targets between countries. As discussed beforehand there have been approaches to broaden outcome measures in terms of medical, care, human resource and other dimensions (8, 12, 130). Yet, policymakers and stakeholders seem to rely on this easily quantifiable indicator to report system performance and to address public expectations regarding emergency services. In some systems, providers may even face financial or regulatory penalties when response time targets are not met. Consequently, practitioners and decision-makers rely on planning and operation of OHEMS systems in a way that facilitates compliance with response time targets.

To investigate the significance of response time in OHEMS, Reuter Oppermann et al. (8) included an analysis in their review. The authors concluded that most studies focus on process quality – according to the Donabedian’s structure, process, and outcome dimensions of care. In expansion of this narrow view, the European Emergency Data project (131) considered five indicators to monitor and evaluate quality:

- Availability,
- Reliable access,
- Demand/workload,
- Rate of critical conditions,
- Level of care.

This common project was the first to incentivize a potential shift from response time. Almost 20 years later, a joint statement by almost 20 EMS domain experts in the United States renewed the call for OHEMS performance measures beyond response time (130). The authors suggested criteria for the following domains:

1. Effective: Is the health care provided clinically appropriate and of high quality?
2. Safe: Are services being provided in a way that is clinically safe for patients, responders, and the community?
3. Satisfying: How do patients and EMS clinicians feel about the service being provided?
4. Equitable: Is the system providing care that is equitable based on patient demographics and service area geography?
5. Efficient: Is this service being provided in a way that maximizes the use of economic and operational resources?

According to their perspective, whenever feasible, evidence-based performance measures should be used that are associated with improved patient outcomes and system performance.

### 4.3 OHEMS systems configurations

Extending on the beforehand perspectives, it also seems important to highlight the influence of contextual factors of the respective OHEMS. For example, the number of available resources and the serviced area may significantly influence scholarly approaches.

Table 5 Comparing Served Population by Ambulances across Different Regions.

**Table 5:** Comparing served population by ambulance stations across different regions.

Location	Serving population	Stations	Citizens served per station	Year	Source/year
Samsun (Turkey)	480.000	4	120.000	2013	Terzi (70)
Oslo and Ankerhuus (Norway)	1.500.000	19	78.000	2023	Schjolberg (50)
Natal (Brazil)	877.000	12	73.000	2021	Dos Santos Cabral (48)
Bavaria (Germany)	13.000.000	484	26.000	2023	Alt (145)
Slovak Republic	5.400.000	274	20.000	2022	Janosikova (32)
Lower Austria (Austria)	1.600.000	158	10.000	2018	Fritze (69)

Note that especially in urban areas ambulance stations may host more than one ambulance as seen in Alt (147) and Fritze (69).

The example of Table 5 shows a range from 1 station/ambulance per 10.000 up to 1 station/ambulance per 120.000 citizens. Subsequently, modeling approaches may range from location optimization approaches in systems that are not yet serving or covering a probable much higher demand (44, 114) up to decision support simulation-optimization models (63), in may have well resourced, but apparently high load systems, that aim to reconfigure existing resources. Although Reuter-Oppermann et al. provided a broad overview and drafted a typical system in their review (8), our review shows that in terms of served population, call volume and coverage area configurations and performance threshold seem to vary considerably.

Furthermore, type of incidents a system is mainly faced with may influence the respective modeling approach. For instance, scholars may focus on optimizing for improved capabilities regarding high speed traffic accidents (51, 132), violent crime incidents (44, 77) or user-abandonment (114), specifically address the issue of high load (87) or the matter of equitable service delivery (110). Other specifics regard the scope of interventions provided, typically described as Basic Life Support, Advanced Life Support, ground or airborne physician staffed units.

### 4.4 The evolving role of OHEMS as healthcare providers

Informed by the findings of this review the authors noted a potential gap between current modeling approaches and OHEMS practice, more specifically regarding the evolving role in becoming a provider of patient centered care (8, 13). Considering the growing academic literature regarding non-conveyance of ambulance clinicians (133–136) and the changing role of paramedic professionals towards becoming healthcare providers (137) it seems that this aspect may be valuable for further consideration. Sikka (1) defined emergency care as “the rapid and appropriate care of victims of traumatic and medical emergencies.” By mentioning the term “appropriate care,” the perspective on professional’s duties extends beyond fast intervention and transport of patients. Modern OHEMS may be conceptualized as a “system of coordinated response and emergency medical care”. As defined by the NHTSA office of EMS they build upon the pillars of public safety, public health, emergency management and healthcare (138).

One practical example of this shift in perception was the introduction of community paramedicine. By implementing these specialists, systems try to better addresses the considerable amount of patients that use ambulances for primary care sensitive problems (139–142), chronic diseases, mental health issues, social issues as well as the repetitive ambulance use—so called frequent users (143, 144). This approach also widens the perspective of interventions towards treatment and referral to mental health or other services (145).

Across the reviewed modeling studies, however, we did not find explicit incorporation of non-conveyance, on-scene treatment, or referral alternatives into formal model structures or objective functions.

### 4.5 Introducing an extended OHEMS Modeling framework

**Figure 7:**
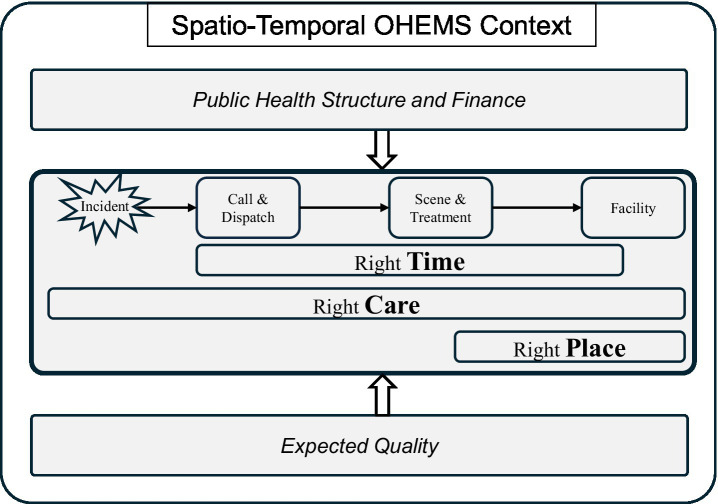
Extended understanding of OHEMS modeling based on right time, right care, right place.

Informed by the synthesis of the presented state of the art in modeling and discussions on the evolving role of OHEMS as a healthcare provider, we propose a possible forward-looking extended understanding of OHEMS modeling (Figure 7). We emphasize that this framework is a conceptual proposal by the authors rather than a direct conclusion drawn from the reviewed models.

The suggested framework highlights the importance of “when” and “where” a modeling study is conducted. Next, the socio-cultural context may be expressed by the public healthcare structure, available financial resources, as well as the expected level of quality by the community. These factors can influence the proposed modeling approach and expectations regarding outcome parameters and represent a vertical trade-off to us. For example, modelers may considering the conflicting issues between expected quality (i.e., improving response time by t-minutes or t-seconds) and the financial costs (i.e., adding resources, increasing workload through relocations or building new ambulance stations).

At the core “right time,” “right care,” and “right place” are suggested as three equally important pillars.

#### 4.5.1 Right time

Right-time decisions should consider empirical-driven, medically sound metrics. These may integrate suggestions from evidence-based treatment guidelines such as cardiac arrest, stroke, sepsis, severe traumatic injuries or heart attacks. Considering urgency levels may also lead to the introduction of different intervention time frames beyond response time. Each of these factors could involve modeling aspects such as prehospital time, door-to-needle time, on-scene time.

#### 4.5.2 Right care

Right care addresses advanced treatment options provided by practitioners (146). In this respect, models could include perspectives such as treatment and discharge on scene or the use of alternative responses. In the field of non-conveyance, also a discharge effect on system load could be modeled.

#### 4.5.3 Right place

Right place considers the patient-centered decision regarding referral. Since hospital transfer may not always be necessary, patients may be referred to general practitioners, care services, or other healthcare providers. Modelers may develop novel approaches addressing facilities beyond hospital for patients groups such as frequent users.

Altogether, Böbel (5) regards these three aspects—“getting the right patient to the right treatment at the right time” as an integrated pillar of OHEMS. Their findings suggest that a needs-based perspective may be beneficial for future modeling approaches.

In summary this framework should encourage modelers and field experts to consider the wider dimension of OHEMS care. The argument here is, that despite the horizontal, time-based axis, ranging from t_(incident)_ to t_(facility)_ and forms of intervention regarding patient care and place, there may also be a vertical axis worth consideration. Here finance and quality oppose each other, creating need for an equilibrium between invested resources and expected service quality.

## 5 Limitations

This scoping review has several limitations that need to be mentioned. First, due to our broad scope and the resulting large number of papers, we needed to split screening and analysis between researchers. To handle this drawback, a comprehensive data extraction template was developed and several discussion sessions amongst reviewers were conducted. Second, the assignment of studies proved especially challenging, when multiple facets of the ECP were addressed. To overcome this issue a second single choice category was integrated and studies were grouped according to the main aim. Yet, it bears mentioning that the idea of the ECP was not to introduce rigid categories but to illustrate the interconnectedness along a pathway. Third, although we used a structured approach to find appropriate search strings including MeSH terms on PubMed, papers on IEEE, Semantic Scholar, and Google Scholar, we have missed publications—especially from other sources and non-English literature. Fourth, we purposely chose a broad understanding of the term “modeling approaches”: since we perceived modeling as a systematic way of representing certain characteristics within systems, the results ranged from statistical approaches up to the creation of digital twins or decision support systems. Fifth, as this study was conducted as a scoping review, the methodological quality of the included studies was not systematically assessed. Although such a task may not be part of a scoping review in general, it seemed important to highlight at least some of the major drawbacks that arose to us during the analysis of studies.

## 6 Conclusion

This scoping review examined the current state of modeling approaches in out-of-hospital emergency medical services. We analyzed which methods were applied frequently, what purposes they were designed to address, and which assumptions underpinned these approaches. We also investigated the implementation perspective of models into practice and investigated the origins and use of response time. Finally, we lied focus on the changing role of OHEMS practice which has been reflected in the modeling sphere.

Based upon the introduction of the Emergency Care Pathway by Aringhieri (13) we found a shift towards a more patient and process-oriented understanding of OHEMS modeling by emphasizing the interrelation between location, relocation, dispatch, routing, forecasting, workforce management and the interplay with the wider healthcare system. The reviewed studies showed that addressing location issues remains core relevant topic among modelers. Recent advancements have occurred particularly in the areas of real-time online optimization decision support systems, dispatch and routing but also workload-related considerations and alternative ways of OHEMS intervention. Increasing model complexity has advanced the development of complex solution heuristics addressing multiple objectives at once. Real-time data availability has brought a more dynamic tactical/operational support towards OHEMS practitioners. In terms of replicability, we found few but recently growing number of studies that provided the model code.

Regarding collaboration with OHEMS practice, our analysis showed that only a limited number of studies mentioned theory—practice exchange. Based upon the idea of a generic modeling framework that integrates iterative work with field experts, findings from our research show that in many cases, collaborations appeared to remain at the level of data provision or communication of model results. Nevertheless, we found examples of continuous integration through strategic healthcare modeling partnerships (26). Future research could investigate on scientific grounds whether there is an association between the number of publications and a corresponding expansion of practically relevant knowledge.

Informed by the reviewed literature, we interpret that modeling studies continue to conceptualize OHEMS primarily as a rapid response and transport service-based notion. Accordingly, time-based performance indicators such as response time remain central to model evaluation. Following this perspective, many optimization models aim to improve time-based indicators. This focus appears to persist despite repeated criticism of time-based metrics in academic literature and repeated calls to extend beyond response time metrics (12, 130). Notably, despite the growing discourse on low-priority calls (139) or non-conveyance interventions (136), we did not identify any specific modeling approach within the included studies that explicitly considered these aspects.

This observation points to a potentially unresolved gap between current OHEMS modeling approaches and ongoing developments in practice. Analytical models may be valuable not only for optimizing individual performance indicators, but also for supporting a broader understanding of OHEMS operations within integrated healthcare systems. Researchers in this field may therefore benefit from addressing the ongoing shift in OHEMS practice through new modeling approaches. Informed by the results of this review, we proposed an extended modeling framework. At its core Right Care, Right Place, and Right Time could provide possible directions for future modeling approaches. This perspective promotes a more systemic understanding of OHEMS and may help extend modeling beyond response-time optimization toward questions of appropriate care, appropriate destination or referral, and clinically meaningful timing. Yet, when defining OHEMS quality it is necessary to consider the current public health structure and balance between expectations and financial resources needed.

## Footnotes

1
https://osf.io/qenvz
