# Supplementary material for: Modeling IN out-of-hospital emergency medical services—a scoping review of approaches and applications
Source: Front Public Health. 2026 Jun 25;14:1825916. doi: 10.3389/fpubh.2026.1825916 (PMC13346243; doi:10.3389/fpubh.2026.1825916)
Supplement: Supplementary file 1 [file Supplementary_file_1.DOCX]

Supplementary Material

Supplement 1 Data Extraction Template


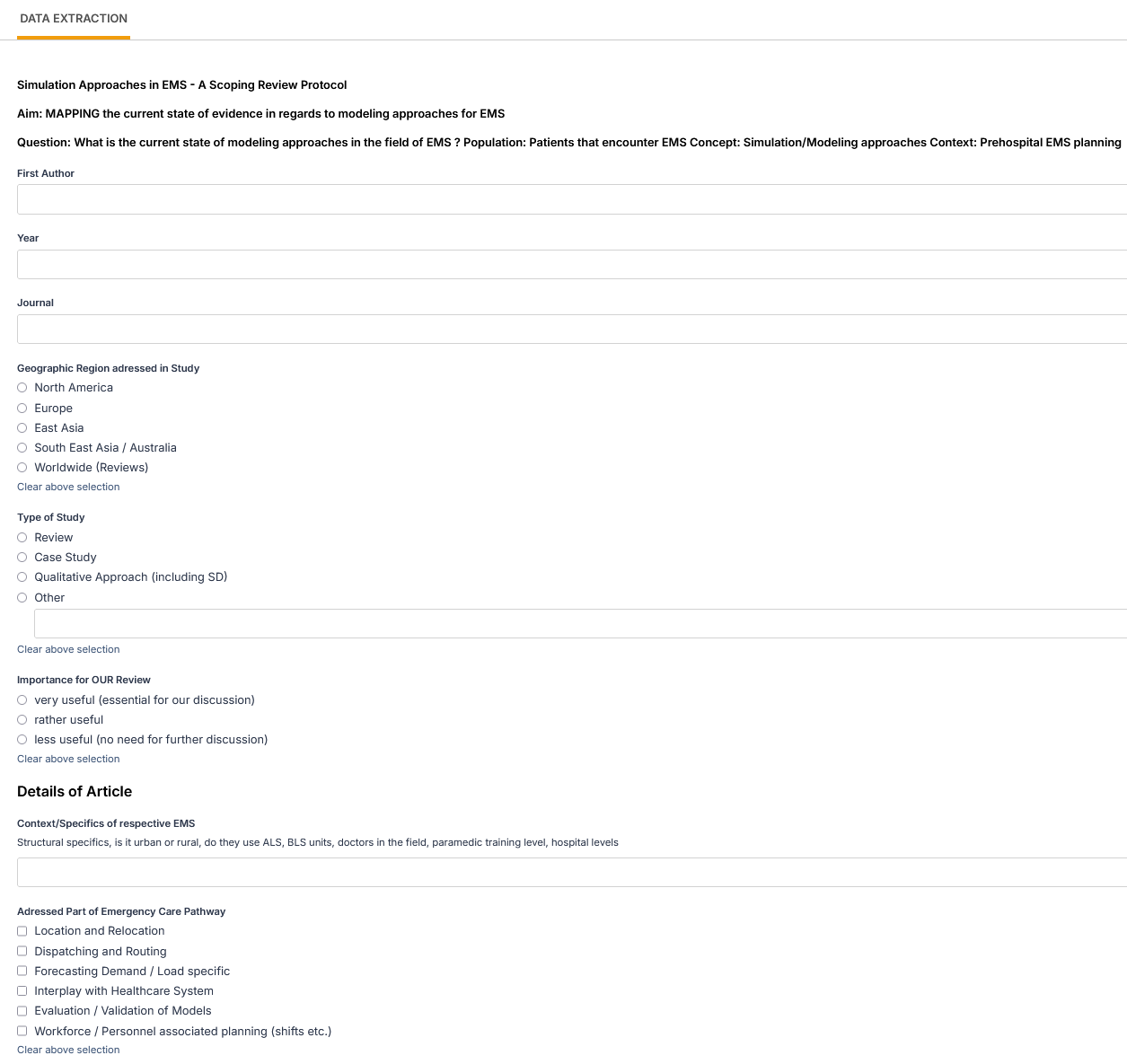


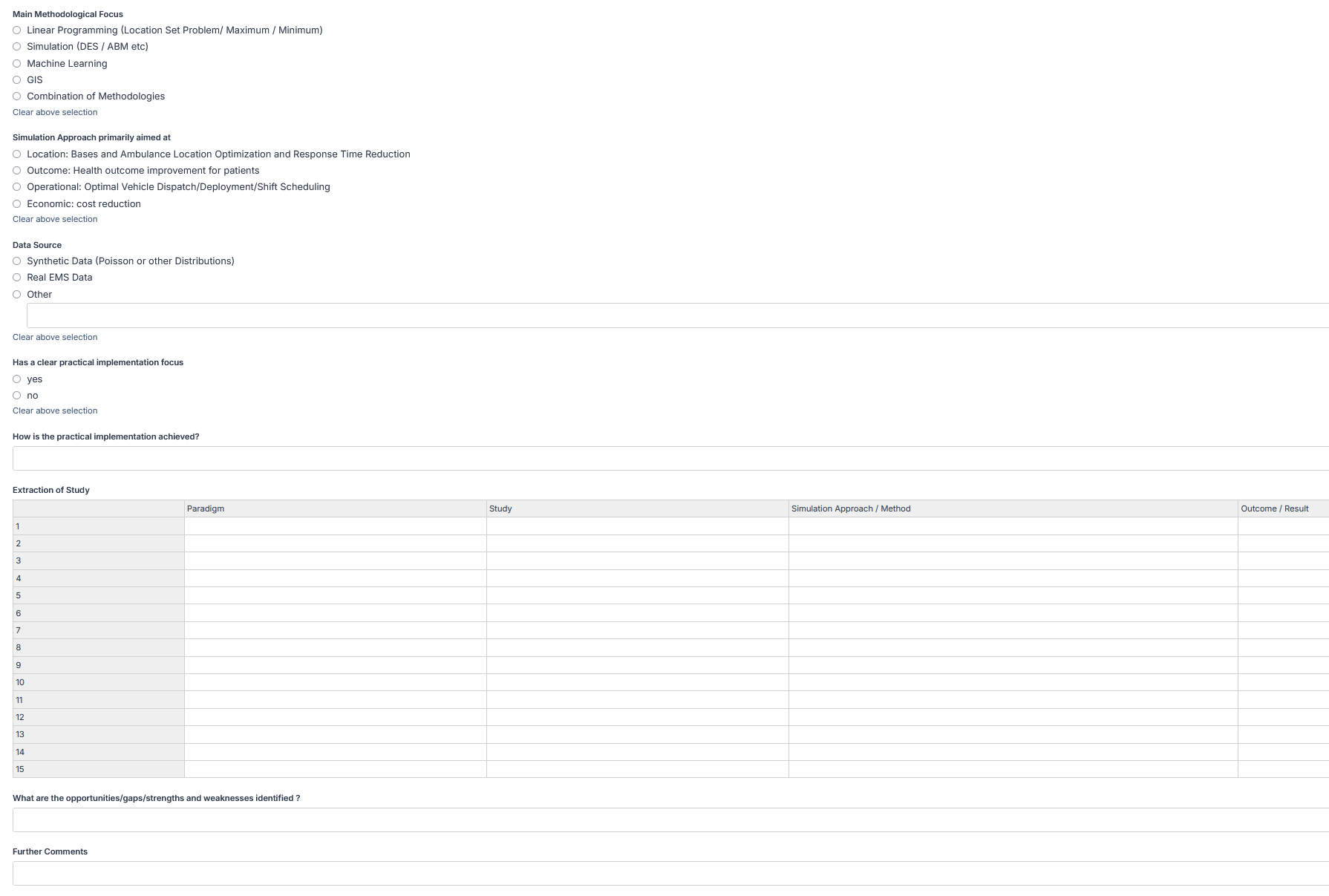


The full list of results from this data extraction can be obtained by the authors.

Supplement 2 Detailed Search Strings of Databases

**Ovid MEDLINE(R) ALL <1946 to January 30, 2025>**

1 exp Systems Analysis/ 109990

2 exp Emergency Medical Services/ 179572

3 exp Ambulances/ 10486

4 1 and 3 82

5 1 and 2 and 3 82

6 5 and 2010:2025.(sa_year). 48

7 exp Emergency Medical Services/ 179572

8 agent based modeling.mp. [mp=title, book title, abstract, original title, name of substance word, subject heading word, floating sub-heading word, keyword heading word, organism supplementary concept word, protocol supplementary concept word, rare disease supplementary concept word, unique identifier, synonyms, population supplementary concept word, anatomy supplementary concept word] 1027

9 7 and 8 4

10 exp Ambulances/ 10486

11 agent based modeling.mp. [mp=title, book title, abstract, original title, name of substance word, subject heading word, floating sub-heading word, keyword heading word, organism supplementary concept word, protocol supplementary concept word, rare disease supplementary concept word, unique identifier, synonyms, population supplementary concept word, anatomy supplementary concept word] 1027

12 10 and 11 0

13 exp Emergency Medical Services/ 179572

14 exp Operations Research/ 71389

15 13 and 14 342

16 Ambulances/ 7251

17 13 and 14 and 16 31

18 17 and 2010:2025.(sa_year). 17

19 exp Emergency Medical Services/ 179572

20 exp Ambulances/ 10486

21 exp Computer Simulation/ 322639

22 19 and 20 and 21 67

23 exp Emergency Medical Services/ 179572

24 exp Ambulances/ 10486

25 optimization.mp. [mp=title, book title, abstract, original title, name of substance word, subject heading word, floating sub-heading word, keyword heading word, organism supplementary concept word, protocol supplementary concept word, rare disease supplementary concept word, unique identifier, synonyms, population supplementary concept word, anatomy supplementary concept word] 224836

26 23 and 24 and 25 62

27 exp Emergency Medical Services/ 179572

28 exp Ambulances/ 10486

29 discrete event simulation.mp. [mp=title, book title, abstract, original title, name of substance word, subject heading word, floating sub-heading word, keyword heading word, organism supplementary concept word, protocol supplementary concept word, rare disease supplementary concept word, unique identifier, synonyms, population supplementary concept word, anatomy supplementary concept word] 1047

30 27 and 28 and 29 16

31 exp Emergency Medical Services/ 179572

32 exp Ambulances/ 10486

33 service times.mp. [mp=title, book title, abstract, original title, name of substance word, subject heading word, floating sub-heading word, keyword heading word, organism supplementary concept word, protocol supplementary concept word, rare disease supplementary concept word, unique identifier, synonyms, population supplementary concept word, anatomy supplementary concept word] 117

34 31 and 32 and 33 5

35 34 and 2010:2025.(sa_year). 2

36 exp Emergency Medical Services/ 179572

37 exp Ambulances/ 10486

38 response time.mp. [mp=title, book title, abstract, original title, name of substance word, subject heading word, floating sub-heading word, keyword heading word, organism supplementary concept word, protocol supplementary concept word, rare disease supplementary concept word, unique identifier, synonyms, population supplementary concept word, anatomy supplementary concept word] 20874

39 36 and 37 and 38 230

40 39 and 2010:2025.(sa_year). 137

41 exp Emergency Medical Services/ 179572

42 exp Ambulances/ 10486

43 demand.mp. [mp=title, book title, abstract, original title, name of substance word, subject heading word, floating sub-heading word, keyword heading word, organism supplementary concept word, protocol supplementary concept word, rare disease supplementary concept word, unique identifier, synonyms, population supplementary concept word, anatomy supplementary concept word] 233685

44 41 and 42 and 43 311

45 44 and 2010:2025.(sa_year). 202

46 exp Emergency Medical Services/ 179572

47 exp Ambulances/ 10486

48 system load.mp. [mp=title, book title, abstract, original title, name of substance word, subject heading word, floating sub-heading word, keyword heading word, organism supplementary concept word, protocol supplementary concept word, rare disease supplementary concept word, unique identifier, synonyms, population supplementary concept word, anatomy supplementary concept word] 87

49 46 and 47 and 48 1

50 49 and 2010:2025.(sa_year). 1

51 exp Emergency Medical Services/ 179572

52 exp Ambulances/ 10486

53 Equity.mp. [mp=title, book title, abstract, original title, name of substance word, subject heading word, floating sub-heading word, keyword heading word, organism supplementary concept word, protocol supplementary concept word, rare disease supplementary concept word, unique identifier, synonyms, population supplementary concept word, anatomy supplementary concept word] 44845

54 51 and 52 and 53 15

55 54 and 2010:2025.(sa_year). 9

56 exp Emergency Medical Services/ 179572

57 exp Ambulances/ 10486

58 routing.mp. [mp=title, book title, abstract, original title, name of substance word, subject heading word, floating sub-heading word, keyword heading word, organism supplementary concept word, protocol supplementary concept word, rare disease supplementary concept word, unique identifier, synonyms, population supplementary concept word, anatomy supplementary concept word] 5921

59 56 and 57 and 58 19

60 59 and 2010:2025.(sa_year). 17

61 exp Emergency Medical Services/ 179572

62 exp Ambulances/ 10486

63 exp Artificial Intelligence/ or exp Machine Learning/ or exp Algorithms/ 493624

64 61 and 62 and 63 121

65 exp Emergency Medical Services/ 179572

66 exp Ambulances/ 10486

67 fleet management.mp. [mp=title, book title, abstract, original title, name of substance word, subject heading word, floating sub-heading word, keyword heading word, organism supplementary concept word, protocol supplementary concept word, rare disease supplementary concept word, unique identifier, synonyms, population supplementary concept word, anatomy supplementary concept word] 32

68 65 and 66 and 67 2

69 exp Emergency Medical Services/ 179572

70 exp Ambulances/ 10486

71 modeling.mp. [mp=title, book title, abstract, original title, name of substance word, subject heading word, floating sub-heading word, keyword heading word, organism supplementary concept word, protocol supplementary concept word, rare disease supplementary concept word, unique identifier, synonyms, population supplementary concept word, anatomy supplementary concept word] 373530

72 69 and 70 and 71 42

73 exp Emergency Medical Services/ 179572

74 exp Ambulances/ 10486

75 forecast*.mp. [mp=title, book title, abstract, original title, name of substance word, subject heading word, floating sub-heading word, keyword heading word, organism supplementary concept word, protocol supplementary concept word, rare disease supplementary concept word, unique identifier, synonyms, population supplementary concept word, anatomy supplementary concept word] 123132

76 73 and 74 and 75 74

77 76 and 2010:2025.(sa_year). 31

78 exp Emergency Medical Services/ 179572

79 exp Ambulances/ 10486

80 ems planning.mp. [mp=title, book title, abstract, original title, name of substance word, subject heading word, floating sub-heading word, keyword heading word, organism supplementary concept word, protocol supplementary concept word, rare disease supplementary concept word, unique identifier, synonyms, population supplementary concept word, anatomy supplementary concept word] 18

81 78 and 79 and 80 4

82 exp Emergency Medical Services/ 179572

83 exp Ambulances/ 10486

84 reposition*.mp. [mp=title, book title, abstract, original title, name of substance word, subject heading word, floating sub-heading word, keyword heading word, organism supplementary concept word, protocol supplementary concept word, rare disease supplementary concept word, unique identifier, synonyms, population supplementary concept word, anatomy supplementary concept word] 28347

85 82 and 83 and 84 4

86 exp Emergency Medical Services/ 179572

87 exp Ambulances/ 10486

88 coverage.mp. [mp=title, book title, abstract, original title, name of substance word, subject heading word, floating sub-heading word, keyword heading word, organism supplementary concept word, protocol supplementary concept word, rare disease supplementary concept word, unique identifier, synonyms, population supplementary concept word, anatomy supplementary concept word] 185213

89 86 and 87 and 88 131

90 exp Emergency Medical Services/ 179572

91 exp Ambulances/ 10486

92 exp Systems Theory/ 3117

93 90 and 91 and 92 3

94 (modeling and ambulance).mp. [mp=title, book title, abstract, original title, name of substance word, subject heading word, floating sub-heading word, keyword heading word, organism supplementary concept word, protocol supplementary concept word, rare disease supplementary concept word, unique identifier, synonyms, population supplementary concept word, anatomy supplementary concept word] 105

95 limit 94 to yr="2010 - 2025" 89

96 hospital.mp. [mp=title, book title, abstract, original title, name of substance word, subject heading word, floating sub-heading word, keyword heading word, organism supplementary concept word, protocol supplementary concept word, rare disease supplementary concept word, unique identifier, synonyms, population supplementary concept word, anatomy supplementary concept word] 1651978

97 (94 and 95) not 96 43

98 exp Ambulances/ 10486

99 exp Models, Theoretical/ 2026996

100 exp Emergency Medical Services/ 179572

101 98 and 99 and 100 449

102 location.mp. [mp=title, book title, abstract, original title, name of substance word, subject heading word, floating sub-heading word, keyword heading word, organism supplementary concept word, protocol supplementary concept word, rare disease supplementary concept word, unique identifier, synonyms, population supplementary concept word, anatomy supplementary concept word] 391397

103 101 and 102 45

104 103 and 2010:2025.(sa_year). 32

105 exp Emergency Medical Services/ 179572

106 exp Ambulances/ 10486

107 exp Markov Chains/ 16804

108 105 and 106 and 107 8

109 108 and 2010:2025.(sa_year). 7

**Semantic Scholar**

"ambulance" AND "agent based modeling" from 2010 to 2024

"ambulance" AND "EMS planning" from 2010 to 2024

"ambulance" AND "forecast" AND "demand" from 2010 to 2024

"ambulance" AND "linear programming" from 2010 to 2024

"ambulance" AND "mathematical programming" from 2010 to 2024

"ambulance" AND "operations research" from 2010 to 2024

"ambulance" AND "repositioning" from 2010 to 2024

"ambulance" AND "simulation" AND "location" from 2010 to 2024

"ambulance" AND "simulation" AND "optimization" from 2010 to 2024

"ambulance" AND "systems theory" from 2010 to 2024

**Google Scholar**

"ambulance" + "Agent based modeling" OR "operations research" OR "mathematical programming" OR "linear programming" OR "monte carlo" -hospital from 2010 to 2024, no citations, no patents, only review articles

**IEEE**

("ambulance" AND("Agent based modeling" OR "operations research" OR "mathematical programming" OR "linear programming" OR "monte carlo" OR "discrete event simulation") )

Supplement 3 Results table with studies from 2018 – 2025 showing steps of the emergency care pathway, study aim, approach, main performance indicator practical implementation.

|  |  |  |  |  |  |  |  |
| --- | --- | --- | --- | --- | --- | --- | --- |
| **ECP** | **First Author** | **Year** | **Main Apporach / Aim** | **Key Aspects** | **KPI** | **Implementation** | **Also Adresses** |
| Location and Relocation | Da Ros | 2024 | Decision Support System for Ambulance Services in Northern Italy | optimizer for automatically suggesting decisions & a simulator to determine the potential outcomes  multi objective optimization for efficiency and fairness  Model publicly available: https://github.com/iolab-uniud/easynet | time based including fairness | yes | Evaluation Validation, Dispatching and Routing |
|  | McHenry | 2024 | Combining GIS and Regression | Incorporation of Equity Aspects - considers whether deprived areas need more care - inverse care law | Deprivation | no | Evaluation Validation |
|  | Zhang | 2024 | Multiperiod Capacitated Facility Location Problem with Maximum Travel Time and Backup Service | Mixed Integer Linear Programming 1) ambulance travel time KPI 2) maximum service capacity 3) dynamic spatio-temporal demand 4) demand coverage 5) backup coverage 6) demand changes due to population mobility | Time based | no |  |
|  | Schjølberg | 2023 | Simulation - Optimization using Dynamic Discrete Event Simulation (DES) and Metaheuristics | Experimental Study of Metaheuristics of location using a Simulation Optimization Approach to improve response time | Time based | no | Evaluation Validation |
|  | Hammami | 2023 | Stochastic chance constraints programming with independent constraints | Model a two-tiered EMS System under demand uncertainty | Time based | no |  |
|  | Jagtenberg | 2023 | Maximum Coverage for Emergency Helicopters | Using population data to reduce response time - using the value of statistical lives and lives needed to save to argue in favor of additional bases | Time based | no |  |
|  | Hashtarkhani | 2023 | Mixed Integer Capacitated Maximum Coverage to capture an acceptable level of calls | Implementation of a relocation of existing and allocation of new vehicles model | Time based | no |  |
|  | Gunnarsson | 2023 | Combining a MCLP and Fringe Sensitive Location Method | Incorporation of fairness by optimizing a weighted sum of coverage, meaning minimizing weighted response time for those not covered withing service standard time | Time based Fairness criterion | no |  |
|  | Frichi | 2022 | Discrete Event Simulation | Including Advanced Life Support (ALS) and Basic Life Support (BLS) ambulance | Time based | no |  |
|  | Uncu | 2022 | Simulation - Optimization Framework using a Discrete Event Simulation | Reallocation of Ambulance Stations for better performance | Time based | no | Evaluation Validation |
|  | Umam | 2022 | Simulation - Optimization using a Metaheuristic Symbiotic Organism Search Algorithm | Change a centralized into a decentralized system for better performance | Time based | no |  |
|  | Majlesinasab | 2022 | Combination of relocation and shortening activation time of ambulances | Using Queing Theory to reduce activation time and applying a MEXCLP and Maximal expected coverage relocation problem | Time based | yes |  |
|  | Ong | 2022 | Integrated Simulation - Optimiziation Framwork, solving two staged stochastic and linear problems | Decision Support tool to optimally location ambulance stations using real data  Model publicly available: https://github.com/joshua-ong/ AmbulanceDeployment | Time based | yes | Evaluation Validation |
|  | Kvet | 2022 | Optimization using a weighted p-median | Denying multiple stations at the same place and including fixed stations | Time based | no |  |
|  | Hatami-Marbini | 2022 | Simulation based optimiziation approach | Multi Objective Model - location of stations, assign the ambulances to maximize survival and minimize costs | Time based survival | yes |  |
|  | Grot #1 | 2022 | Incorporating Fairness in location planning | Including Rawlsian criterion - maximizing coverage of the least covered and GINI coefficient - minimize differences between covered areas | Time based Fairness criterion | no |  |
|  | Gloabian #1 | 2022 | Repositioning of ambulances according to system status | Hypercube queuing model - multiverse optimizer Multi-Verse Optimizer and Genetic algorithms to maximize coverage probability | Time based | no |  |
|  | Strauss | 2022 | Event Based Simulation for Dynamic Ambulance Redeployment | Integrating a discrete event simulator as well asdynamicc reinforcement learning to gain the optimal redeployment strategy for ambulances | Time based | no |  |
|  | Zaheeruddin | 2022 | Shuffled Frog Leaping Algorithm (mSFLA) to provide an optimal allocation plan | Comparison of the Shuffled Frog Leaping Algorithm with Genetic Algorithm and Particle Swarm Optimization | Time based | no | Evaluation Validation Forecast |
|  | Janisokova | 2022 | Implementing a hierarchical p-median model and a Discrete Event Simulation | Nationwide implementation in both urban and rural contexts for Slovak Republic | Time based | yes | Evaluation Validation |
|  | de Graaf | 2021 | Population based Helicopter Location | Using a simulated annealing based algorithm | Time based | no |  |
|  | Jagtenberg | 2021 | Different Equality measures of helicopter locations | Social Welfare Function, Bernoulli Nash, Iso-elastic Social Welfare Function questioning the current practice of optimizing base locations for efficiency only | Fairness criterion | no |  |
|  | Jankovic | 2021 | Modular capacitated location model using simulation as validation | A modular capacitated location model outperforms p-median, MEXCLP and expected response time models | Time based | no |  |
|  | Janosikova | 2021 | Bi-criteria mathematical programming model using Simulation as validation | Access for high priority patients within short time and average response time | time based | no | Evaluation Validation |
|  | Kochetov | 2021 | Simulation Optimization Model | Genetic Algorithm Optimizer and Discrete Event Simulation | Time based | no |  |
|  | Zhu | 2021 | GIS based Spatial accessibility of Emergency Medical Services for elderly | Bayesian Kriging to get Spatial Equilibrium | Accessibility  Distance | no |  |
|  | Lee | 2021 | Combining GIS and Optimization | developing a GIS based backup coverage model | Time based | no | Evaluation Validation |
|  | Smedley | 2021 | Using GIS to calcuate isochrones around hospitals | By lowering the mission ground time more coverage is possible | Time based | no |  |
|  | Carbral | 2021 | Metaheuristic using a Genetic Algorithm for a Double Standard Model | Model allows a backup if the ambulance is occupied | Time based | no |  |
|  | Strauss | 2021 | Rule based discrete event simulation for EMS | Using historic incident data, creating a historical scenario, then modifying parameters (resources, bases) and simulating under new circumstances | Time based | yes | Evaluation Validation Dispatch and Routing |
|  | Eckman | 2020 | Infinitesimal perpetuation Analysis (IPA) gradient estimator | Gadient-based search on an ambulance base location problem | Time based | no |  |
|  | Catumba-Ruiz | 2020 | Hybrid Genetic Algorithm and a Discrete event simulation | Optimize response time, effective assigment of resources, satisfaction of demand, usage of vehicles | Time based Demand based Satisfaction of Demand | no | Dispatch and Routing Evaluation and Validation  Workforce |
|  | Stone | 2020 | Integrating elliptical flight distances into modeling | More realistic view | Time based | no |  |
|  | Andersson | 2020 | Decision Support Tool using a maximum Expected Performance Location Problem for Heterogeous Regions (MEPLP-HR) | Testing the results of a closure of a local hospital, the implementation of a designated non-urgent transport vehicle, time varying demand and resources | Time based | no |  |
|  | Carbral | 2019 | Optimization using a weighted p-median | Minimize traveled distance for units with 7 bases for the city in accordance with city planner | Time based | yes |  |
|  | Kvet | 2019 | Fast Approximate Algorithm | fast algorithm for robust rescue system design, which enables to obtain a good approximate solution of the original problem in a short time | Time based | no |  |
|  | Yang | 2019 | Simulation based optimization framework | Guassian Mixutre Model to describe demand distribution, simulation models can never provide an optimal solution - output of the simulation is being sent to the optimization | Time based | no |  |
|  | van den Berg | 2019 | Maximum Expected Covering Location Model | Green and Brownfield analysis | Time based | no |  |
|  | Trujillo | 2019 | Robust Double Standard Model with Relocation | Depending on the preferred outcome (maximize coverage or reduced relocation missions, minimize number of bases used) DSM-R, RDSM or mDSM might prove beneficial | time based | no |  |
|  | Valencia-Nunez | 2018 | Probablistic Model for Arrival Times based on location | Using a GIS and Monte Carlo simulation for minimizing implementation costs on ambulance stations | time based | no |  |
|  | Li | 2018 | Maximum Coverage using a double standard model | Include relocation suggestions and backup coverage | time based | no |  |
|  | Aringhieri | 2018 | Real Time Simulation and Optimization of Ambulances | maximize the number of emergency requests served within a time threshold, and to minimize the waiting times | time based | no |  |
|  | Caelho | 2018 | Kriging-based metamodeling simulation optimization | Kringing to minimize average response time and comparison with OptQuest | time based | no |  |
|  | Fritze | 2018 | Maximum Coverage location problem for physician staffed ambulance cars | Increment number of relocated bases to see the effects | time based | yes |  |
| Dispatching and Routing | Herche | 2024 | Selecting the appropriate ambulance based on case urgency instead of next available, preserving ambulances for life threatening emergencies | prioritize the vehicle that minimizes this risk among all ambulances whose dispatch would increase the response time by less than a given threshold | time based prioritization | no |  |
|  | Bhatia | 2024 | Simulation - optimization using a Negamax algorithm for optimizing the dispatch of ambulances during emergencies | Creating a smart ambulance system containing 1) Data collection and Processing, 2) Analysis and Prediction 3) Resource management and Optimization 4) Communication and User Interface 5) Storage using real time data, having a predictive component, using multi factors and creating a scalable platform for city authorities | time based | no | Evaluation Validation  Forecast |
|  | Luan | 2024 | Vehicle routing optimization for Highway Incidents | mixed Linear Problems with semi soft time windows MIPSSTW | time based | no |  |
|  | Elfahim | 2022 | Combining Reinforcement learning and deep neural networks | Using Markov decision process to improve dispatch of ambulances to faster reach patients | time based | no |  |
|  | Torres | 2021 | Machine Learning for a realistic depiction of estimated travel times of ambulances | using a random forest model to get a realistic depiction of travel times as suggested by OSRM and other Routing tools | time based | no |  |
|  | Shin | 2020 | Patient prioritization after mass incident event | finite-horizon Markov Decision Process | time based | no |  |
|  | Hua | 2020 | Optimal dispatch via reinforcement learning | using a Markov decision process to implement a dispatch strategy that minimizes average response time | time based | no |  |
|  | Pınarbasi | 2020 | Optimal transport strategies in post and pre-disaster periods | combining Voroni diagrams and a heuristic approach to minimize maximal completion time for injured after a disaster | time based | no |  |
|  | Hojgaard | 2020 | Estimate travel time on GPS basis | provide a simple equation of travel time between a GPS predicted and actual arrival time | time based | no |  |
|  | Marla | 2020 | Maximum Likelihood Estimator including a simulation-based optimization to reduce ambulance abandonment | Improve tactical short term dispatch decisions as well as long term strategic location decisions. Abandonment increases with waiting times for an ambulance | time based  Willingness to wait | no | Evaluation Validation |
|  | Roa | 2020 | Matheuristic for online real time relocation problem | ambulance dispatch, relocation using a double standard model (backup-location) and a heuristic to maximize preparedness | time based system preparedness | no | location allocation evaluation Validation |
|  | Memari | 2020 | bi-objective dynamic location-helicopter ambulance allocation-ambulance routing model with multi-medical server | Integration of temporary ambulance stations  the case of disaster response | time based | no | location allocation  Evaluation Validation |
|  | Lam | 2019 | Open Source Cloud Based Tracking and Optimization Decision Support Tool | Optimize bases, track EMS vehicles, develop a customizable dispatch simulator  Model publicly available: https://github.com/EMSTrack/EMSTrack-Docker | time based | yes | Location and Allocation  Evaluation Validation |
|  | Park | 2019 | Simulation Optimization Model for a two tiered (ALS/BLS) ambulance system considering errors in patient severity classification | Optimal ambulance operation policy using a model that includes patient severity classification errors using survival function raises ethical questions | time based  survival rate | no | Evaluation Validation |
|  | Schneider | 2018 | Simulation of travel time estimation when using lights and sirens | Calculate isochrones for stations using the adapted estimated speed profile | time based driving time | no | Evaluation Validation |
|  | Fu | 2018 | Game theory coupled with a simulation to develop a city wide dispatch model for the Case of Acute Coronary Disease | distribute patients over hospital beds equally to provide resources | time based availability of hospital beds | no | Interplay with Healthcare System Evaluation Validation |
|  | Lanzarone | 2018 | Recursive Simulation - Optimiziation Approach to solve the Ambulance Location Dispatching Problem | Aiming to improve the busy fraction approximation  Developing a recursive Optimization-Simulation Approach (ROSA) | time based | no |  |
|  | Amorim | 2018 | Agent based model to test dispatching rules | testing closest idle vs. survival vs. random | time based survival | no |  |
| Forecast | Manaa | 2024 | Using Machine Learning for ambulance coverage prediction | Using different prediction models to improve coverage | time based | no |  |
|  | Xu | 2024 | Simulation based Forecasting model for hospital bed availability | Decide row 1, column Simulation Approach / MethodThe ABFS predicts the conditions in various EDs to understand why they reject some patients and admit others, and the information gained will be used to connect all ERs using a cloud-based simulation system | availability of hospital beds | yes | Interplay with Healthcare System  Dispatch and Routing |
|  | Naess | 2024 | Machine Learning for buysiness assessment of ambulances | Connecting the delay of ambulances with the overall busyness | time based | no |  |
|  | Rezaei | 2024 | Spatio-temporal incident forecasting for tactical EMS planning | Showing the effects of different forecasting methods (moving average, TBATS, seasonality) Model publicly available: https://github.com/joshua-ong/ | spatio- temporal incidents | yes |  |
|  | Monks | 2023 | Timeseries Forecasting of Ambulance Demand | Ensemble of models (Arima, harmonic regression, TBATS, Seasonality) outperforms a single model  Implementation in South West England Model publicly available: https://doi.org/10.5281/zenodo.4850149 | spatio-  temporal incidents | yes |  |
|  | Nicoletta | 2022 | Spatio temporal prediction of ambulance demand | Bayesian hierarchical model (generalized linear mixed effects model for time dependent areal data) approach to predict | spatio- temporal incidents | no |  |
|  | Wong | 2022 | Forecasting ambulance demand using weather data | Using a autoregressive integrated moving average model ARIMA | spatio temporal incidents | no |  |
|  | Walker | 2021 | Machine Learning to predict ambulance patient door to off strechter waiting times | Linear regression vs. random forest vs elastic net regression | time based | no |  |
|  | Lin | 2020 | Next Day Prediction of Ambulance calls Using several state of the art methods | Regional Moving Average, Linear Regression, Support Vector Regression, Multi Layer Perception, Light GBM | time based | no |  |
|  | Karmal | 2020 | Vehicle future location prediction | NextSTMove algorithm to predict the next location of an ambulance vehicle | location | no |  |
|  | Granberg | 2018 | Near future state of EMS vehicle prediction | Using a discrete event simulation the possible future states of the EMS system are predicted to select the appropriate ambulance | time based | no | Evaluation Validation |
| Evaluation / Validation | Strauss | 2024 | Lessons from a decade of modeling using a Discrete Event Simulation model | Mathematical Modeling seems to be more for academic research, Simulation more towards practical implementation because of its versatility - Modeling should go hand in hand with system analysis | time based | yes |  |
|  | MohdHassan | 2023 | Dava envelopment analysis to measure efficiency of ambulances | Efficiency as the maximum feasible output from given input (cost) and output (distance) | distance | no |  |
|  | Begicheva | 2020 | Combining a System Dynamics and an Agent Based Model to evaluate the current ems system | integrating a stocks and flow model considering when emergencies appear, testing the results on three dimensions: time based, workload and patient satisfaction | time based workload patient satisfaction | no | Interplay with Healthcare System |
| Workforce | Sariyer | 2023 | Newsvendor approach to optimize capacity in an ems callcenter | minimizing the total excepted underage and overage costs |  | no |  |
| Interplay with Healthcare System | Maas | 2023 | Monte carlo simulation | Drip and ship (bring the patient to the specials) vs. drive the doctor (bring the specialist to the patient) for thrombectomy | time based | no | Evaluation Validation |
|  | Pilberry | 2023 | Co-Modeling Discrete Event Simulation | Triaging 999 (EMS) into 111 (Primary Care) in case the problem is primary care sensitive Model publicly available: https://github. com/RichardPilbery/MOOOD-study | triaging | yes | Evaluation Validation |
|  | Wolf | 2022 | Digital Twin for Smart City for multi agency response | Real time monitoring and control, optimizing workflow and providing predictive and preventive maintenance | time based | no | Forecast |
|  | Phan | 2019 | Mathematical approach to locate mobile stroke units and compare the impact to hospitals | Model publicly available: https://gntem3.shinyapps.io/ambmc | time based | no | Interplay with healthcare system |
|  | Kovalchuk | 2018 | Combining Game theory and modeling approach | Modeling confronting / contradicting stakeholder positions for ambulance use (overcrowding) | stakeholder positions | no | Evaluation Validation |
|  | Pforringer | 2018 | Agent Based Simulation of Hospital Closure Policies on Ambulance Diversion | Using the IVENA Patient Monitoring System and test policies on diversion and their effects  seeing the significant impact of EMS on ER workload (~20% in Munich) | time based closure time periods | yes | Evaluation Validation |

Supplement 4 Links and Repositories for Replication of Models

| Study | Title | Link |
| --- | --- | --- |
| Da Ros, 2024 | Supporting Fair and Efficient Emergency Medical Services in a Large Heterogeneous Region | <https://github.com/iolab-uniud/easynet> |
| Lam 2019 | Low-Cost Open-Source Solution to Optimize Emergency Medical Services in Developing Communities by Tracking, Dispatching, and Simulating | <https://github.com/EMSTrack/> |
| Monks 2023 | Forecasting the daily demand for emergency medical ambulances in England and Wales: a benchmark model and external validation. | <https://github.com/TomMonks/swast-forecast-tool> |
| Monks 2023 | Forecasting the daily demand for emergency medical ambulances in England and Wales: a benchmark model and external validation. | <https://github.com/TomMonks/swast-benchmarking>  <https://hub.docker.com/r/tommonks01/swast-benchmark> |
| Ong 2022 | Open EMS: an open-source Package for Two-Stage Stochastic and Robust Optimization for Ambulance Location and Routing with Applications to Austin-Travis County EMS Data | <https://github.com/joshua-ong/AmbulanceDeployment> |
| Phan, 2017 | Googling Service Boundaries for Endovascular Clot Retrieval Hub Hospitals in a Metropolitan Setting | <https://github.com/GNtem2/AmbMC>  <https://gntem3.shinyapps.io/ambmc/> |
| Pilbery 2023 | Modelling NHS England 111 demand for primary care services: a discrete event simulation. | <https://github.com/RichardPilbery/MOOOD-study> |

**Repositories of unpublished or only referenced Papers**

<https://github.com/uoa-ems-research/JEMSS.jl?tab=readme-ov-file>

<https://github.com/niklasdbs/ambusim>

<https://github.com/fschuetz04/simcpp20?tab=readme-ov-file>

**Repositories of Generic Location Optimization Frameworks**

<https://pysal.org/spopt/index.html>

<https://github.com/HIGISX/hispot/tree/main>

<https://github.com/cyang-kth/maximum-coverage-location>

<https://github.com/mitre/ffoaal/tree/main>

Supplement 5 – Comprehensive Study Table

| # | Year | First Author | Title |
| --- | --- | --- | --- |
| 1 | 2024 | Luan | Does the priority of ambulance guarantee no delay? a MIPSSTW model of emergency vehicle routing optimization considering complex traffic conditions for highway incidents. |
| 2 | 2024 | Heche | Inhomogeneous Poisson Process for Ambulance Dispatch |
| 3 | 2024 | Ouertani | The Dynamic Vehicle Routing Problem: A Comprehensive Survey |
| 4 | 2024 | Sukmawati | Vehicle Routing Problem in Under Deadlines: A Systematic Literature Review |
| 5 | 2024 | Xu | The emergency medical service dispatch recommendation system using simulation based on bed availability. |
| 6 | 2024 | Strauss | Insights from a Decade of Optimizing Emergency Medical Services Across Three Major Regions in Switzerland |
| 7 | 2024 | Næss | Using machine learning to assess the extent of busy ambulances and its impact on ambulance response times: A retrospective observational study. |
| 8 | 2024 | Manaa | Application of machine learning techniques for ambulance coverage prediction |
| 9 | 2024 | Rezaei | Forecasting to support EMS tactical planning: what is important and what is not. |
| 10 | 2024 | Zhang | A multi-period capacitated facility location problem with maximum travel time and backup service for locating and sizing EMS stations |
| 11 | 2024 | McHenry | Equity in the provision of helicopter emergency medical services in the United Kingdom: a geospatial analysis using indices of multiple deprivation. |
| 12 | 2024 | Belfragea | Simulating change: A systematic literature review of agent-based models for policy-making |
| 13 | 2024 | Bhatia | Enhancing Emergency Response: A Smart Ambulance System Using Game-Building Theory and Real-Time Optimization |
| 14 | 2023 | Maroof | Vehicle Routing Optimization for Humanitarian Supply Chain: A Systematic Review of Approaches and Solutions |
| 15 | 2023 | Hassan | Data envelopment analysis for ambulance services of different service providers in urban and rural areas in Ministry of Health Malaysia |
| 16 | 2023 | Monks | Forecasting the daily demand for emergency medical ambulances in England and Wales: a benchmark model and external validation. |
| 17 | 2023 | Mass | ‘Drive the doctor’ for endovascular thrombectomy in a rural area: a simulation study |
| 18 | 2023 | Pilbery | Modelling NHS England 111 demand for primary care services: a discrete event simulation. |
| 19 | 2023 | Jagtenberg | Utopia for Norwegian helicopter emergency medical services: Estimating the number of bases needed to radically bring down response times, and lives needed to be saved for cost effectiveness. |
| 20 | 2023 | Schjølberg | Comparing Metaheuristic Optimization Algorithms for Ambulance Allocation: An Experimental Simulation Study |
| 21 | 2023 | Hammami | A Chance Constrained Stochastic Programming Model for Designing Two-tiered Emergency Medical Service Systems |
| 22 | 2023 | Gunnarsson | Locating helicopter ambulance bases in Iceland: efficient and fair solutions. |
| 23 | 2023 | Hashtarkhani | Where to place emergency ambulance vehicles: use of a capacitated maximum covering location model with real call data. |
| 24 | 2023 | Becker | Dynamic ambulance relocation: a scoping review. |
| 25 | 2023 | Da Ros | Supporting Fair and Efficient Emergency Medical Services in a Large Heterogeneous Region |
| 26 | 2022 | Kong | EMS Operations Management: Simulation, Optimization, and New Service Models |
| 27 | 2022 | Elfahim | Deep Reinforcement Learning Approach for Emergency Response Management |
| 28 | 2022 | Ceklic | Ambulance dispatch prioritisation for traffic crashes using machine learning: A natural language approach. |
| 29 | 2022 | Nicoletta | Bayesian spatio-temporal modelling and prediction of areal demands for ambulance services |
| 30 | 2022 | Wong | Forecasting daily emergency ambulance service demand using biometeorological indexes. |
| 31 | 2022 | Wolf | Towards a digital twin for supporting multi-agency incident management in a smart city. |
| 32 | 2022 | Majlesinasab | Performance evaluation of an EMS system using queuing theory and location analysis: A case study. |
| 33 | 2022 | Uncu | An improved EMS simulation-optimization model with Poisson mixture distribution |
| 34 | 2022 | Zaheeruddin | Optimally Allocating Ambulances in Delhi using Mutation based Shuffled Frog Leaping Algorithm |
| 35 | 2022 | Golabian1 | A multi-verse optimizer algorithm for ambulance repositioning in emergency medical service systems |
| 36 | 2022 | Kvet | Optimization of Emergency Medical Service with Fixed Centers |
| 37 | 2022 | Grot | Fairness or efficiency-Managing this conflict in emergency medical services location planning |
| 38 | 2022 | Umam1 | Simulation Optimization for Location and Allocation of Emergency Medical Service |
| 39 | 2022 | Neira-Rodado | Ambulances Deployment Problems: Categorization, Evolution and Dynamic Problems Review |
| 40 | 2022 | Frichi | Assessing and improving ambulance coverage in the prefecture of Fez using discrete-event simulation |
| 41 | 2022 | Ong | OpenEMS: an open-source Package for Two-Stage Stochastic and Robust Optimization for Ambulance Location and Routing with Applications to Austin-Travis County EMS Data |
| 42 | 2022 | Marbini | An emergency medical services system design using mathematical modeling and simulation-based optimization approaches |
| 43 | 2022 | Strauß | A Comparison of Ambulance Redeployment Systems on Real-World Data |
| 44 | 2022 | Janosikova | Reorganization of an Emergency Medical System in a Mixed Urban-Rural Area. |
| 45 | 2021 | Lee | Multi-agent reinforcement learning algorithm to solve a partially-observable multi-agent problem in disaster response |
| 46 | 2021 | Torres | Correction of the travel time estimation for ambulances of the red cross Tijuana using machine learning. |
| 47 | 2021 | Walker | Predicting Ambulance Patient Wait Times: A Multicenter Derivation and Validation Study. |
| 48 | 2021 | Grot | Enhanced coverage by integrating site interdependencies in capacitated EMS location models. |
| 49 | 2021 | Lee | Statewide Ambulance Coverage of a Mixed Region of Urban, Rural and Frontier under Travel Time Catchment Areas. |
| 50 | 2021 | Kochetov | Optimization of the Ambulance Fleet Location and Relocation |
| 51 | 2021 | Smedley | How Does Mission Ground Time Impact on Population Coverage of Aeromedical Retrieval Systems?. |
| 52 | 2021 | Janosikova | Coverage versus response time objectives in ambulance location. |
| 53 | 2021 | Jagtenberg | Introducing fairness in Norwegian air ambulance base location planning. |
| 54 | 2021 | de Graaf | Optimising base locations for New Zealand's Helicopter Emergency Medical Services. |
| 55 | 2021 | Jankovic | Ambulance Locations in a Tiered Emergency Medical System in a City |
| 56 | 2021 | Golabian | A simulation-optimization algorithm for return strategies in emergency medical systems |
| 57 | 2021 | Ghobadi | Integration of Facility Location and Hypercube Queuing Models in Emergency Medical Systems |
| 58 | 2021 | Strauss | Optimizing Emergency Medical Service Structures Using a Rule-Based Discrete Event Simulation—A Practitioner’s Point of View |
| 59 | 2021 | Zhu | Spatial Accessibility Assessment of Prehospital EMS with a Focus on the Elderly Population: A Case Study in Ningbo, China. |
| 60 | 2020 | Hua | Optimal Dispatch in Emergency Service System via Reinforcement Learning |
| 61 | 2020 | Pınarbasi | On the Evaluation of the Ambulance Capacity of the Asian Side of Istanbul in the Case of a Serious Earthquake. |
| 62 | 2020 | Shin | A Meta Algorithm for Reinforcement Learning: Emergency Medical Service Resource Prioritization Problem in an MCI as an Example |
| 63 | 2020 | Højgaard | Response time of emergency vehicles may be predicted using ordinary GPS estimates. |
| 64 | 2020 | Mercuur | Integrating social practice theory in agent-based models: A review of theories and agents |
| 65 | 2020 | Marla | Managing EMS systems with user abandonment in emerging economies |
| 66 | 2020 | Lin | Leveraging Machine Learning Techniques and Engineering of Multi-Nature Features for National Daily Regional Ambulance Demand Prediction. |
| 67 | 2020 | Kamal | Future Location Prediction for Emergency Vehicles Using Big Data: A Case Study of Healthcare Engineering. |
| 68 | 2020 | Stone | Aeromedical retrieval of trauma patients: Impact of flight path model on estimates of population coverage. |
| 69 | 2020 | Eckman | Biased Gradient Estimators in Simulation Optimization |
| 70 | 2020 | Tassone | A Comprehensive Survey on the Ambulance Routing and Location Problems |
| 71 | 2020 | Cabral | Metaheuristics in the decentralization of SAMU bases using simulation in northeastern Brazil. |
| 72 | 2020 | Roa | An online real-time matheuristic algorithm for dispatch and relocation of ambulances |
| 73 | 2020 | Catumba-Ruiz | A hybrid optimization method for reallocation of mobile resources |
| 74 | 2020 | Memari | Air and ground ambulance location-allocation-routing problem for designing a temporary emergency management system after a disaster. |
| 75 | 2020 | Anderssona | Using optimization to provide decision support for strategic emergency medical service planning - Three case studies. |
| 76 | 2020 | Begicheva | Donabedian Approach for Simulation Modeling to Evaluate the Quality of Emergency Medical Services in a Large Metropolitan Area: A Case Study |
| 77 | 2019 | Schneider | Simulation-Based Location Optimization of Ambulance Stations |
| 78 | 2019 | Park | Two-Tiered Ambulance Dispatch and Redeployment considering Patient Severity Classification Errors. |
| 79 | 2019 | Kvet | Fast Approximate Algorithm for Robust Emergency System Design |
| 80 | 2019 | Trujillo | Comparative analysis of relocation strategies for ambulances in the city of Tijuana, Mexico. |
| 81 | 2019 | Itani | Ambulance allocation models: A review |
| 82 | 2019 | van den Berg | Improving ambulance coverage in a mixed urban-rural region in Norway using mathematical modeling. |
| 83 | 2019 | Eric Lucas dos Santos Cabral | Optimization model for the installation of SAMU bases: application in Natal-RN |
| 84 | 2019 | Yang | Simulation modeling and optimization for ambulance allocation considering spatiotemporal stochastic demand |
| 85 | 2019 | Baxter | Quantitative modeling in disaster management: A literature review |
| 86 | 2019 | Lam | Low-Cost Open-Source Solution to Optimize Emergency Medical Services in Developing Communities by Tracking, Dispatching, and Simulating |
| 87 | 2019 | Aringhieri | Online optimization in health care delivery: Overview and possible applications |
| 88 | 2019 | Phan | Googling Boundaries for Operating Mobile Stroke Unit for Stroke Codes |
| 89 | 2018 | Kovalchuk | Towards Model-Based Policy Elaboration on City Scale Using Game Theory: Application to Ambulance Dispatching |
| 90 | 2018 | Amorim | Emergency Medical Service Response: Analyzing Vehicle Dispatching Rules |
| 91 | 2018 | Guo | Measuring Impact of Emerging Transportation Technologies on Community Equity in Economy, Environment and Public Health or Equity Assessment for … |
| 92 | 2018 | Granberg | SIMULATION BASED PREDICTION OF THE NEAR-FUTURE EMERGENCY MEDICAL SERVICES SYSTEM STATE |
| 93 | 2018 | Pförringer | Closure simulation for reduction of emergency patient diversion: a discrete agent-based simulation approach to minimizing ambulance diversion. |
| 94 | 2018 | Fritze | Combining spatial information and optimization for locating emergency medical service stations: A case study for Lower Austria |
| 95 | 2018 | Aringhieri | A SIMULATION AND ONLINE OPTIMIZATION APPROACH FOR THE REAL-TIME MANAGEMENT OF AMBULANCES |
| 96 | 2018 | Li | A Maximal Covering Location Model of Ambulances in Emergency Medical Service |
| 97 | 2018 | Valencia-Nuñez | Probabilistic Model for Managing the Arrival Times of Pre-Hospital Ambulances Based on their Geographical Location (GIS) |
| 98 | 2018 | Coelho | Kriging-based simulation optimization: An emergency medical system application |
| 99 | 2018 | Cabral | Response time in the emergency services. Systematic review. |
| 100 | 2018 | Lanzarone | A RECURSIVE OPTIMIZATION-SIMULATION APPROACH FOR THE AMBULANCE LOCATION AND DISPATCHING PROBLEM |
| 101 | 2018 | Belanger | Recent optimization models and trends in location, relocation, and dispatching of emergency medical vehicles |
| 102 | 2018 | Fu | UvA-DARE ( Digital Academic Repository ) Coupling Game Theory and Discrete-Event Simulation for Model-Based Ambulance |
| 103 | 2018 | Alotaibi | Agent-Based Simulation for Coordination Emergency Response: A Review Study |
| 104 | 2017 | Lam | Simulation-based decision support framework for dynamic ambulance redeployment in Singapore. |
| 105 | 2017 | Tlili | A mathematical model for efficient emergency transportation in a disaster situation. |
| 106 | 2017 | Çapar | Alternative metrics to measure EMS system performance |
| 107 | 2017 | Laker | Understanding Emergency Care Delivery Through Computer Simulation Modeling |
| 108 | 2017 | Shahriari | Bi-objective approach for placing ground and air ambulance base and helipad locations in order to optimize EMS response. |
| 109 | 2017 | Mota | Optimization and simulation of an ambulance location problem |
| 110 | 2017 | Garner | Locating helicopter emergency medical service bases to optimise population coverage versus average response time. |
| 111 | 2017 | Dibene | Optimizing the location of ambulances in Tijuana, Mexico. |
| 112 | 2017 | Reuter-Oppermann | Logistics for Emergency Medical Service systems |
| 113 | 2017 | Orlov | Assessment of ambulance station efficiency at congested city traffic |
| 114 | 2017 | Aringhieri | Emergency medical services and beyond: Addressing new challenges through a wide literature review |
| 115 | 2017 | Sariyer | Sizing capacity levels in emergency medical services dispatch centers: Using the newsvendor approach. |
| 116 | 2016 | Jagtenberg | Dynamic ambulance dispatching: is the closest-idle policy always optimal?. |
| 117 | 2016 | Daglayan | An Optimized Ambulance Dispatching Solution for Rescuing Injures after Disaster |
| 118 | 2016 | Lujak | Distributed coordination of emergency medical service for angioplasty patients |
| 119 | 2016 | Chen | Demand Forecast Using Data Analytics for the Preallocation of Ambulances. |
| 120 | 2016 | Oberscheider | Analysis of the impact of different service levels on the workload of an ambulance service provider. |
| 121 | 2016 | Lui | Review and prospect of studies on emergency management |
| 122 | 2016 | Zaffar | Coverage, survivability or response time: A comparative study of performance statistics used in ambulance location models via simulation–optimization |
| 123 | 2016 | Bélanger | An empirical comparison of relocation strategies in real-time ambulance fleet management |
| 124 | 2016 | Krishnan | Robust ambulance allocation using risk-based metrics |
| 125 | 2016 | Lee | Iterative optimization algorithm with parameter estimation for the ambulance location problem. |
| 126 | 2016 | van den Berg | Comparison of static ambulance location models |
| 127 | 2016 | Lahijanian | Double coverage ambulance location modeling using fuzzy traveling time |
| 128 | 2016 | Bozorgi-Amiri | Integrated locating of helicopter stations and helipads for wounded transfer under demand location uncertainty. |
| 129 | 2016 | Aringhieri | Supporting decision making to improve the performance of an Italian Emergency Medical Service |
| 130 | 2015 | Widener | Ground and Helicopter Emergency Medical Services Time Tradeoffs Assessed with Geographic Information. |
| 131 | 2015 | Sreekanth | Equity-constrained dispatching models for emergency medical services |
| 132 | 2015 | van Buuren | A simulation model for emergency medical services call centers |
| 133 | 2015 | Stein | Meeting national response time targets for priority 1 incidents in an urban emergency medical services system in South Africa: More ambulances won't help. |
| 134 | 2015 | Kao | The Impact of Ambulance and Patient Diversion on Crowdedness of Multiple Emergency Departments in a Region. |
| 135 | 2015 | Degel | Time-dependent ambulance allocation considering data-driven empirically required coverage. |
| 136 | 2015 | McCormack | A simulation model to enable the optimization of ambulance fleet allocation and base station location for increased patient survival |
| 137 | 2015 | McQueen | Enhanced care team response to incidents involving major trauma at night: are helicopters the answer?. |
| 138 | 2015 | García | Covering location problems |
| 139 | 2014 | Bandara | Priority dispatching strategies for EMS systems |
| 140 | 2014 | Kleinscheg | Improving emergency medical dispatching with emphasis on mass-casualty incidents. |
| 141 | 2014 | Boyacı | Hypercube Queueing Models for Emergency Response Sys- tems |
| 142 | 2014 | Cantwell | Ambulance demand: random events or predicable patterns?. |
| 143 | 2014 | Lutter | Analysis of Ambulance Location Models Using Discrete Event Simulation |
| 144 | 2014 | Zhen | A simulation optimization framework for ambulance deployment and relocation problems |
| 145 | 2014 | Lam | Dynamic ambulance reallocation for the reduction of ambulance response times using system status management. |
| 146 | 2014 | Fakhimi | A hybrid agent-based and Discrete Event Simulation approach for sustainable strategic planning and simulation analytics |
| 147 | 2013 | Pillac | A review of dynamic vehicle routing problems |
| 148 | 2013 | Nouman | Developing a Distributed Agent-Based and DES Simulation Using poRTIco and Repast |
| 149 | 2013 | Cerquides | A tutorial on optimization for multi-agent systems |
| 150 | 2013 | Anagnostou | Distributed hybrid agent-based discrete event emergency medical services simulation |
| 151 | 2013 | Delgado | Reducing ambulance diversion at hospital and regional levels: systemic review of insights from simulation models |
| 152 | 2013 | Morohosi | Optimization model and simulation for improving ambulance service system |
| 153 | 2013 | Terzi | A geographic information system-based analysis of ambulance station coverage area in Samsun, Turkey. |
| 154 | 2013 | Khodaparasti | A new combined dynamic location model for emergency medical services in fuzzy environment |
| 155 | 2013 | Nogueira | Reducing Emergency Medical Service response time via the reallocation of ambulance bases. |
| 156 | 2013 | Luo | A new model for planning emergency facilities in Shanghai |
| 157 | 2013 | Diao | A Literature Review on the Optimization Method of Emergency Transportation and Logistics System |
| 158 | 2013 | Cox | Optimizing a Drone Network to Respond to Opioid Overdoses |
| 159 | 2013 | Ingolfsson | EMS Planning and Management |
| 160 | 2013 | Aboueljinane | A review on simulation models applied to emergency medical service operations |
| 161 | 2013 | Mason | Simulation and Real-Time Optimised Relocation for Improving Ambulance Operations |
| 162 | 2012 | Doumouras | Comparing Methodologies for Evaluating Emergency Medical Services Ground Transport Access to Time-critical Emergency Services: A Case Study Using Trauma Center Care. |
| 163 | 2012 | Lin | Managing emergency department overcrowding via ambulance diversion: a discrete event simulation model. |
| 164 | 2012 | Wong | Weather factors in the short-term forecasting of daily ambulance calls. |
| 165 | 2012 | Lee | A simulation-based iterative method for a trauma center — Air ambulance location problem |
| 166 | 2012 | Basar | A taxonomy for emergency service station location problem |
| 167 | 2012 | Ni | Exploring bounds on ambulance deployment policy performance |
| 168 | 2012 | Azizan | Application of OpenStreetMap Data in Ambulance Location Problem |
| 169 | 2012 | Farahani | Covering problems in facility location: A review |
| 170 | 2012 | van Buuren | Evaluating dynamic dispatch strategies for emergency medical services: TIFAR simulation tool |
| 171 | 2012 | Guaracao | Optimizing Resources Involved in the Reception of an Emergency Call |
| 172 | 2012 | Aboueljinane | Reducing ambulance response time using simulation: The case of Val-de-Marne department Emergency Medical service |
| 173 | 2011 | Ramirez-Nafarrate | Design of centralized Ambulance Diversion policies using Simulation-Optimization |
| 174 | 2011 | Chanta | Improving emergency service in rural areas: a bi-objective covering location model for EMS systems |
| 175 | 2011 | Schmid | Solving the dynamic ambulance relocation and dispatching problem using approximate dynamic programming. |
| 176 | 2011 | Gonzalez | EMS relocation in a rural area using a geographic information system can improve response time to motor vehicle crashes. |
| 177 | 2010 | Wong | Weather inference and daily demand for emergency ambulance services. |
| 178 | 2010 | McLeod | Matching capacity to demand: a regional dashboard reduces ambulance avoidance and improves accessibility of receiving hospitals. |
| 179 | 2010 | Maxwell | Identifying effective policies in approximate dynamic programming: Beyond regression |
| 180 | 2010 | Sasaki | Using genetic algorithms to optimize current and future health planning--the example of ambulance locations. |
| 181 | 2010 | Berchi | A five steps methodology for ambulance planning |
| 182 | 2010 | Hock Ong | Reducing ambulance response times using geospatial-time analysis of ambulance deployment. |
| 183 | 2010 | Silva | Emergency medical systems analysis by simulation and optimization |
